# Intermediate Cu(II)-Thiolate Species in the Reduction of Cu(II)GHK by Glutathione: A Handy Chelate for Biological Cu(II) Reduction

**DOI:** 10.1021/acs.inorgchem.1c02669

**Published:** 2021-11-15

**Authors:** Iwona Ufnalska, Simon C. Drew, Igor Zhukov, Kosma Szutkowski, Urszula E. Wawrzyniak, Wojciech Wróblewski, Tomasz Frączyk, Wojciech Bal

**Affiliations:** † Chair of Medical Biotechnology, Faculty of Chemistry, Warsaw University of Technology, Noakowskiego 3, Warsaw 00-664, Poland; ‡ Polish Academy of Sciences Institute of Biochemistry and Biophysics, Pawińskiego 5a, Warsaw 02-106, Poland; § Department of Medicine (Royal Melbourne Hospital), The University of Melbourne, Victoria 3010, Australia; ∥ NanoBioMedical Centre, Adam Mickiewicz University, Wszechnicy Piastowskiej 3, Poznań 61-614, Poland

## Abstract

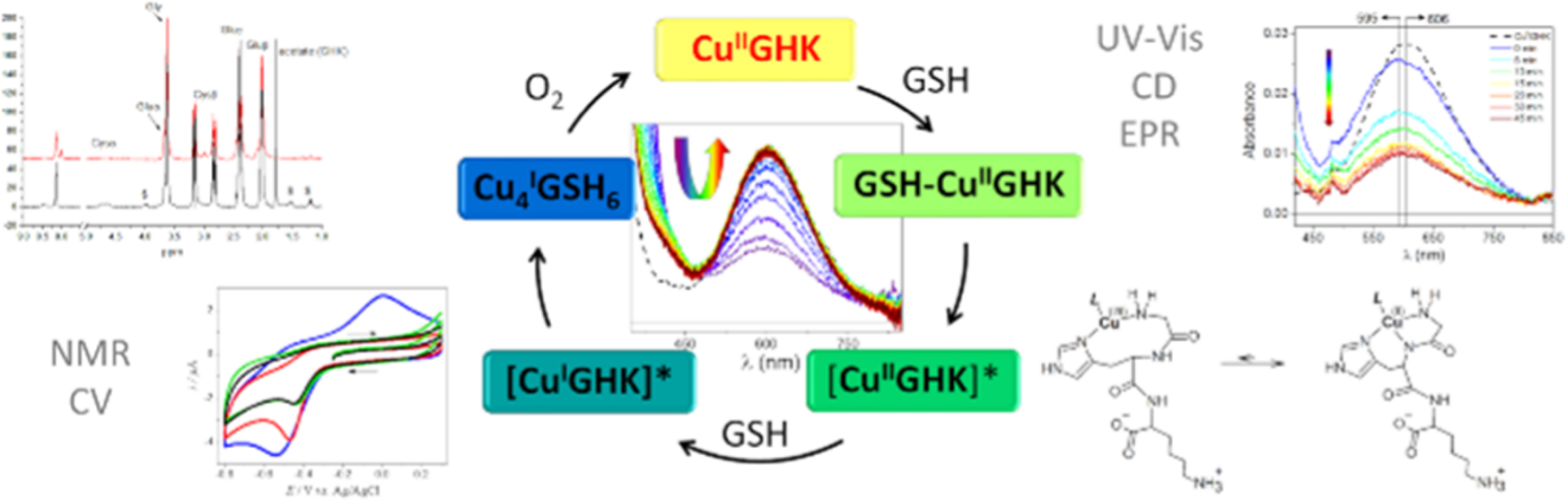

Gly-His-Lys (GHK) is a tripeptide present in the human bloodstream that exhibits a number of biological functions. Its activity is attributed to the copper-complexed form, Cu(II)GHK. Little is known, however, about the molecular aspects of the mechanism of its action. Here, we examined the reaction of Cu(II)GHK with reduced glutathione (GSH), which is the strongest reductant naturally occurring in human plasma. Spectroscopic techniques (UV–vis, CD, EPR, and NMR) and cyclic voltammetry helped unravel the reaction mechanism. The impact of temperature, GSH concentration, oxygen access, and the presence of ternary ligands on the reaction were explored. The transient GSH-Cu(II)GHK complex was found to be an important reaction intermediate. The kinetic and redox properties of this complex, including tuning of the reduction rate by ternary ligands, suggest that it may provide a missing link in copper trafficking as a precursor of Cu(I) ions, for example, for their acquisition by the CTR1 cellular copper transporter.

## Abstract 2

Gly-His-Lys (GHK) is a human bioactive tripeptide thought to be activated by Cu(II) binding, but little is known about the molecular aspects of its action. UV−vis, circular dichroism (CD), EPR, and NMR spectroscopies, and cyclic voltammetry were used to examine the reduction of Cu(II)GHK with glutathione (GSH), the most abundant biological thiol. A semistable GSH-Cu(II)GHK reaction intermediate was discovered, with properties suitable for delivering Cu(I) to biological transport proteins.

## Introduction

Gly-His-Lys (GHK) is a high-affinity copper chelator naturally occurring in human blood (log ^c^*K*_7.4_ = 12.62).^1^ The origin of the tripeptide is still not clear. It is most likely released through proteolytic degradation of extracellular proteins, such as SPARC (secreted protein acidic and rich in cysteine) or type I collagen, in response to the process of tissue damage.^2,3^ GHK was discovered in 1973 by L. Pickart and M. Thaler, who noted that liver tissue from an elderly donor treated with blood plasma from a young volunteer regained the ability to produce proteins specific to the young tissue.^4^ This rejuvenating effect was attributed to a higher level of GHK since a decline in the tripeptide concentration with age has been noted (from 200 ng mL^–1^ at the age of 20 to 80 ng mL^–1^ by the age of 60).^5^ GHK was isolated from blood plasma as a Cu(II) complex. This suggested the involvement of Cu(II) in its biological activity, which includes stimulation of growth and proliferation of various cultured cell types, leading to tissue regeneration, wound healing, angiogenesis, radiation damage recovery, and even anxiety reduction in laboratory animals.^6−14^ The skin regenerating activity, stimulation of collagen synthesis, and anti-inflammatory, antioxidant, and anti-aging properties^5,15^ prompted the widespread use of Cu(II)/GHK in skin care (due to the often limited knowledge on specific complexes, we used a slash in the formula where the components are known, but they may represent various stoichiometries or the exact stoichiometry is not known). As a regulator of expression of a number of genes,^16^ it has been proposed as a therapeutic agent for certain cancers, chronic obstructive pulmonary disease (COPD), and bleomycin-induced pulmonary fibrosis.^17−20^ The mechanistic aspects of this multitude of activities remain, however, obscure, and it is tempting to speculate that they include copper handling on the tissue level.

The binary Cu(II)GHK complex was extensively studied using spectroscopic methods, potentiometry, calorimetry, and crystallography.^1,21−27^ The Cu(II) chelation occurs via the glycine amino nitrogen, the deprotonated amide nitrogen of the Gly–His peptide bond, and the His side-chain imidazole nitrogen, yielding two fused chelate rings. In this respect, GHK is a representative of the class of H_2_N–Xxx–His (XH) peptides, known as particularly efficient Cu(II) chelators.^28,29^ Importantly, the fourth equatorial binding site in the Cu(II)GHK structure is available for ternary complex formation (here noted as L–Cu(II)GHK, where L represents a monodentate ligand).^1,21,27^ The ability to bind a transient partner and a fast Cu(II) exchange rate between the holo and apo forms reinforced a postulated Cu(II) carrier role of GHK, consistent with oxidative conditions in blood.^30^ Indeed, the copper-binding sites in known copper carriers (e.g., human serum albumin, HSA, and cellular copper transporter Ctr1), belonging to the amino-terminal Cu and Ni binding (ATCUN) family,^31^ strongly prefer Cu(II). However, the transfer of copper to cells requires its prior reduction to Cu(I) due to the specificity of the Ctr1 transmembrane channel.^32^ While the site(s) and mechanism(s) of this reduction in humans are a matter of debate,^33^ the GHK participation would require a facile mechanism of reduction of GHK-bound Cu(II) to Cu(I). Previous studies showed that Cu(II)GHK is inert versus the physiological levels of ascorbate,^27^ the major reducing agent in blood.^34^ Nevertheless, body fluids are rich in other antioxidants, such as retinol, α-tocopherol, β-carotene, and reduced glutathione (GSH), protecting against free radicals.^35^ In our studies, we focused on GSH, which is a stronger reductant than ascorbate.^36,37^

GSH (γ-glutamyl-cysteinyl-glycine) is the most abundant thiol in nature.^38^ It is present in millimolar concentrations in most human cells and plays a crucial role in cellular redox homeostasis. Its extracellular level in healthy humans is micromolar.^39,40^ Apart from being a reductant, GSH is also a complexing agent for heavy metals, participating in their cellular detoxication via its thiolate and, additionally, the nitrogen and oxygen donors.^41^ The reaction between GSH and Cu(II) cations includes a swift Cu(II) reduction, yielding Cu(I)/GSH and Cu(II)GSSG complexes, depending on the metal-to-ligand ratio and oxygen availability.^42−45^ The reaction is slowed down for strongly chelated Cu(II), as in complexes with products of enzymatic hydrolysis of Aβ peptides, but with the same copper reduction products.^42^

In this work, we targeted the redox behavior of Cu(II)GHK in the presence of glutathione using UV–vis and circular dichroism (CD) spectroscopies, aided by EPR, NMR, and electrochemical studies. The model ATCUN peptide, C-terminally amidated GGH, was used as a reference, representing biorelevant Cu(II) complexes bearing the saturated 4N coordination plane (such as in HSA and Ctr1). The results allowed us to discuss the mechanism of glutathione-mediated reduction of GHK-bound Cu(II) and its putative physiological role.

## Results and Discussion

The reaction of Cu(II)GHK with GSH was initially monitored by electronic spectroscopy. The addition of 1 mM GSH to the sample containing 0.50 mM GHK with 0.45 mM Cu(NO_3_)_2_ in 50 mM HEPES, pH 7.4, 37 °C, under aerobic conditions resulted in an immediate d–d band decrease, accompanied by an increase of absorbance in the 250–350 nm region. These changes were reverting over a longer period, as revealed by time-dependent measurements (Figure S1).

Next, the effect of experimental conditions on the reaction was studied by comparing its course in 50 mM phosphate buffer, 50 mM HEPES, and unbuffered (water) solution, each at pH 7.4. The comparison of the kinetic traces for selected wavelengths, provided in Figure S2, revealed that each buffer affected the course of the reaction, although the effects of HEPES were subtler. To keep the studied system unambiguous, all further experiments were conducted in pure water. This could be done because, once the sample was prepared, its pH remained stable within 0.2 pH unit during the experiment.

**Figure 1:**
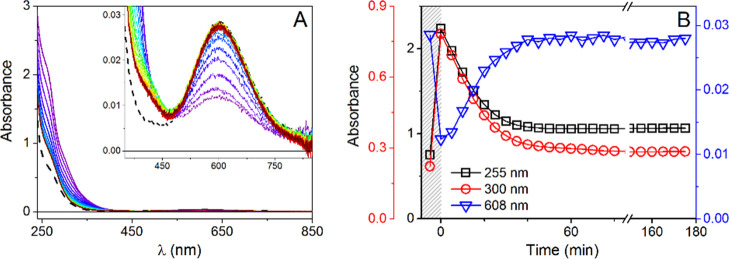
(A) UV–vis spectra collected with 5 min intervals for 0.50 mM GHK with 0.45 mM Cu(II) in the presence of 1.0 mM GSH at pH = 7.4, *T* = 37 °C and (B) absorbance changes at 255 nm (black squares), 300 nm (red circles), and 606 nm (blue triangles), plotted as a function of time; absorbance values for the binary complex are presented in the shaded field; dashed line represents the spectrum of Cu(II)GHK.

The reaction at 37 °C in water, monitored by UV–vis and CD spectra, is presented in Figures 1A and S3, respectively. The CD bands in the vis and near-UV range exhibited only a rapid decrease after GSH addition, followed by a gradual return of the spectral intensity, whereas the UV–vis spectral changes also involved new bands appearing in the near-UV range. A slight blue shift of the d–d band from the initial 606 nm to ca. 595 nm was also noted. Wavelengths for kinetic tracing were selected according to the major intensity differences revealed by differential spectra (Figure S4). In UV–vis, the decrease of the main d–d band was accompanied by the immediate rise of absorbance at 300 and 255 nm, assigned to S-to-Cu(I) charge-transfer (CT) transitions in a Cu(I)/GSH thiolate complex (Figure 1).^46^ The low-intensity band at 410 nm, emerging later in the reaction, originated from the interaction of Cu(II)/Cu(I) with GSH in the presence of oxygen, as confirmed by the control experiments (Figure S5) as well as reported previously.^42^

Then, the kinetic profile of the reaction at 37 °C was studied by single-wavelength measurements at 606 nm to improve its time resolution from 5 min to 5 s (Figure S6). This allowed us to detect the d–d band intensity minimum between the second and fourth minute of observation, which separated the Cu(II) reduction and Cu(I) re-oxidation phases of the reaction.

For the 2.2-fold excess of GSH used in these experiments, the Cu(II) reduction process was incomplete, typically reaching 60%, despite the fact that just 1 mol. equiv. of GSH is required to reduce Cu(II) to Cu(I). Full Cu(II) reduction was achieved at the 4.4- and 6.6-fold GSH excess (2 and 3 mM, Figure S7). At these GSH concentrations, the recovery of the Cu(II)GHK complex also slowed down but was not prevented. Repetitions of the reduction/re-oxidation cycle by periodic additions of adequate GSH amounts indicated a high reproducibility of the examined process (Figure S8). The availability of ambient oxygen was the limiting factor of the re-oxidation step, as demonstrated in Figure S9, analogously to the previously studied ATCUN systems. The Cu(I)/GSH complex is stable under anaerobic conditions.^42,47^

The HPLC separation of reaction products collected after the re-oxidation step, followed by ESI-MS analysis, allowed us to identify GSSG as the only covalent product of the studied reaction (signals at 613.2 and 307.1 *m/z* represent [M + H]^+^ and [M + 2H]^2+^ forms, respectively). No MS-detectable covalent GHK modifications appeared in the reaction (as revealed by the presence of *m/z* = 341.2 for [M + H]^+^ GHK species and the absence of identifiable products of its covalent modification), in agreement with the reproducibility of the reaction cycle (Figures S10 and S11).

Spectroscopic measurements did not indicate a contribution of the Cu(II)GSSG binary complex (characterized by an absorption band at 625 nm^36,42,48^) to the overall Cu(II) equilibrium at the completion of copper re-oxidation. This was expected based on the respective affinity constants at pH 7.4 for Cu(II)GHK versus Cu(II)GSSG, log *K* = 12.62 versus 10.37.^1,48^ The control titration of 0.45 mM Cu(II)GHK with up to 5 mM GSSG did not alter the d–d band position of the former, additionally confirming the absence of any ternary GSSG-Cu(II)GHK species (Figure S12).

**Figure 2:**
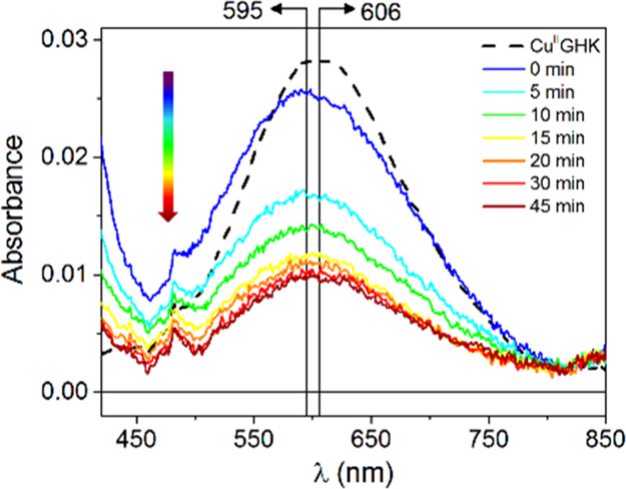
Selected UV–vis spectra collected with 5 min intervals for 0.50 mM GHK with 0.45 mM Cu(II) in the presence of 1.0 mM GSH at 5 °C, pH = 7.4, demonstrating the d–d band blue shift concomitant with the absorbance decrease; the dashed line represents the spectrum of Cu(II)GHK; restoration of the signal for the same reaction is presented in Figure S14B.

Having thus demonstrated that the re-oxidation reaction consisted of oxidation of the Cu(I)/GSH complex into GSSG and Cu(II) ions, which were promptly scavenged by GHK to re-form Cu(II)GHK, we followed the reduction step in greater detail. First, we repeated the reaction at lower temperatures, 20 and 5 °C, whose results are summarized in Figure S13. The reduction phase at 5 °C is presented in detail in Figure 2. The reduction endpoint, measured by the minimum of the d–d absorption peak and the maximum of CT bands, increased from 60% at 37 °C to 70% at 5 °C, while the reaction rate, measured as the time required to reach the minimum, decreased from 3 min to 15 min to 45 min, for 37, 20, and 5 °C, respectively. The re-oxidation phase was also correspondingly slower.

The variation of d–d band position became clearly visible at 5 °C (Figure 2). During the reduction step, a prominent blue shift of the signal from 606 nm down to 595 nm occurred. This lasted for about 10 min and was followed by a partial backshift to ca. 600 nm. This observation strongly suggested that two different phenomena occurred in this system in the given time window. The presence of an additional spectral component at the very beginning of the reaction was further supported by the analysis of differential spectra (Figure S14A). The absorbance decrease around 615 nm parallel to the increase around 505 nm indicated two overlapping Cu(II) species, with one being formed at the expense of another (Figure S14A, inset). These changes were accompanied by a transient band at ca. 345 nm. Such phenomena were not seen in the re-oxidation phase (Figure S14B). Therefore, a short-lived species was characteristic only for the reduction phase.

To further confirm the observed spectral effects that could be misinterpreted due to a low signal-to-noise ratio, another experiment, with 4-fold higher concentrations of reagents, was carried out (Figure S15). Not only the significant blue shift of the d–d band was confirmed but also a profound effect of air oxygen supply on the kinetic profile of the reaction was demonstrated (Figure S15A–C). The increase in the concentration of the binary complex and GSH with limited oxygen access influenced the redox balance in the examined redox system, resulting in the inhibition of the reoxidation phase, as revealed by a comparison to previous results (Figure S15D).

The observed d–d band blue shift indicated that the Cu(II)-coordinated water molecule in Cu(II)GHK was replaced by a stronger field ligand. Large blue shifts (typically 40–60 nm) were observed in 3 + 1N ternary Cu(II) complexes formed by GHK and other XH-type peptides with imidazole rings, with much smaller shifts observed for 3N + 1O complexes.^1,49−51^ An intermediate shift detected here (10–12 nm) could be due to a partial formation of a 3 + 1N ternary species with another GHK molecule, GHK-Cu(II)GHK,^1^ as a consequence of an increased excess of GHK over Cu(II) ions, depleted by partial reduction to Cu(I).

**Figure 3:**
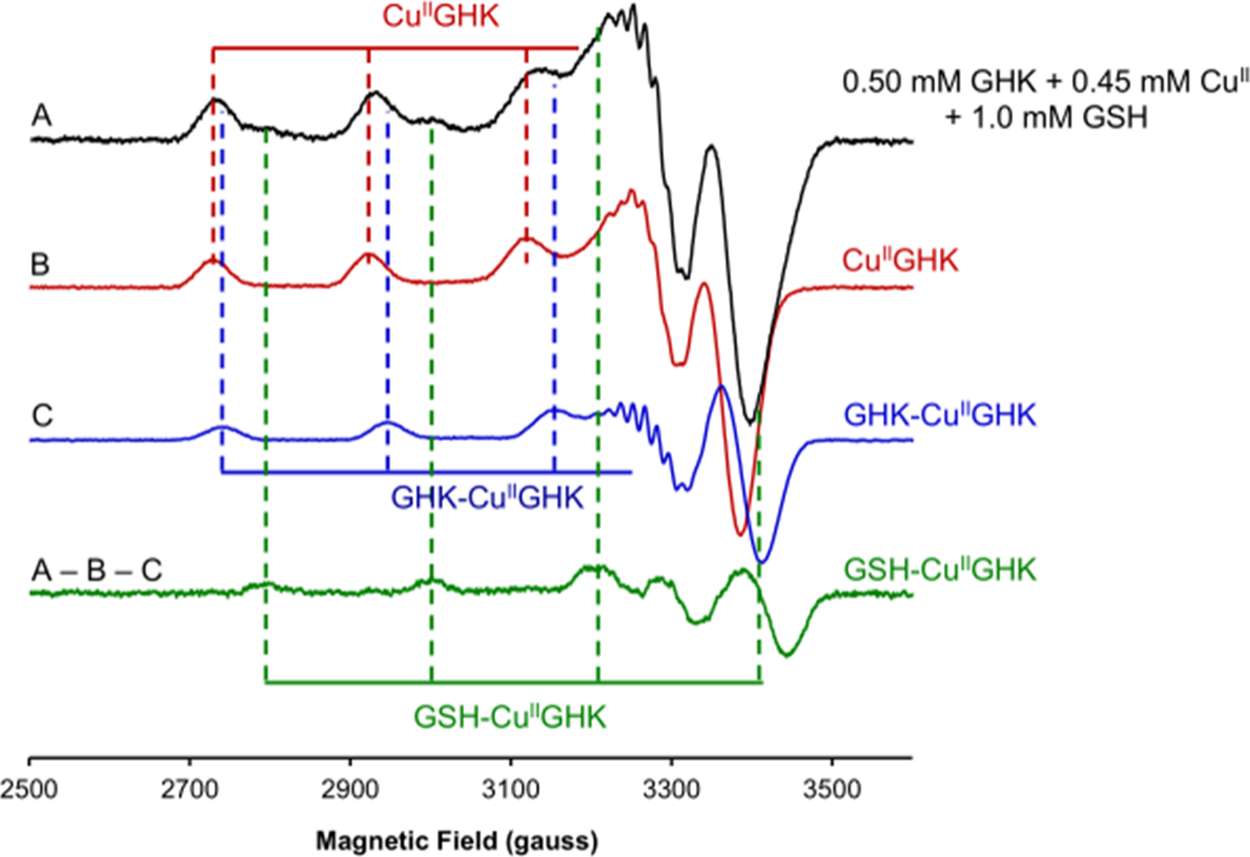
X-band (9.42 GHz) frozen solution (77 K) EPR spectra showing the isolation of the spectrum corresponding to GSH-Cu(II)GHK by weighted subtraction of the spectrum of Cu(II)GHK (Figure S16) and GHK–Cu(II)GHK (Figure S17) from the spectrum obtained for 0.50 mM GHK with 0.45 mM Cu(II) in the presence of 1.0 mM GSH at 4 °C. Dashed vertical lines indicate the approximate position of the prominent Cu(II) hyperfine features of each coordination mode as a guide to the eye.

Alternatively, these spectral changes could result from the engagement of GSH thiolate in the ternary complex, as in previous studies, no evidence was found for the participation of amino acid or peptide amines in 3 + 1N ternary complexes with XH peptides.^1,49−52^ The key evidence for the GSH thiolate binding was provided by EPR measurements taken at given time points for the sample stored on ice. The stepwise isolation of individual signals (Figures S16 and S17) revealed the presence of a short-lived GSH-Cu(II)GHK species within the first minutes of reaction in addition to the Cu(II)GHK and GHK–Cu(II)GHK complexes (Figure 3). The Cu(II)GHK and GHK–Cu(II)GHK spectra were obtained from controls in the absence of GSH, while that of GSH-Cu(II)GHK was derived from the first spectrum of the reaction. The shift of the g parallel value upon GSH addition (from 2.233 to 2.175, Table S2), as well as the change within the superhyperfine structure, is very similar to that observed for analogous thiosemicarbazone complexes, where GSH is bound equatorially to a tridentate ligand^53,54^ It was further affirmed by the consistency of the simulated and experimental EPR data (Figure S18 and Table S2). Using these data, the time course of the reaction on ice was reconstructed in Figure S19, with the full spectral analysis provided in Figure S20. It should be emphasized, however, that freezing the samples for EPR measurement alters the relative binding affinities of binary and ternary species, and according to our previous experience, the apparent stability of GHK–Cu(II)GHK is much higher at 77 K than at room temperature.^1^ This freezing artifact will be investigated in detail and presented elsewhere. On the other hand, the swiftness of the examined reaction required the measurement in a frozen solution mode. Therefore, the EPR-based species distribution ought to be regarded as qualitative but firm evidence for the transient GSH-Cu(II)GHK species.

Despite the uncertain speciation of Cu(II) complexes, EPR provided a reliable quantitation of total Cu(II) (Figure S19), which, along with the published stability constants for Cu(II)GHK and GHK–Cu(II)GHK,^1^ allowed us to calculate the concentrations of these complexes corresponding to the UV–vis spectra at given reaction times. This, in turn, was the basis for the decomposition of d–d signals collected at the beginning of the reaction performed at 5 °C. This analysis, presented in Figure S21, revealed the third Cu(II) component with the d–d maximum at ∼570 nm, which declined over time. This finding correlates well with a transient signal at 345 nm revealed by differential analysis and the short-lived GSH-Cu(II)GHK species detected by EPR.

**Figure 4:**
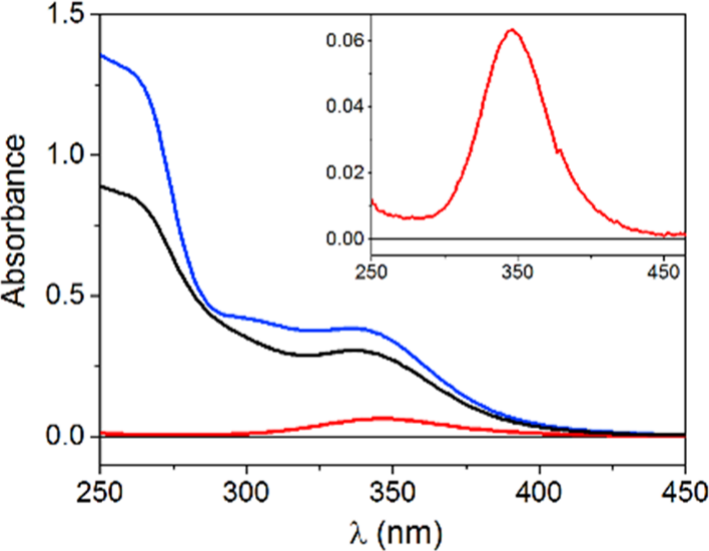
Differential absorption spectra calculated for 0.50 mM GHK with 0.45 mM Cu(II) in the presence of 2.0 mM 2-mercaptoethanol, *T* = 10 °C (black line), 1.0 mM NaSCH_3_, *T* = 15 °C (red line), and 1.0 mM GSH, *T* = 5 °C (blue line) expressed as a difference between the first spectrum recorded after thiol introduction and Cu(II)GHK spectrum.

To learn more about the mechanism of formation of thiolate ternary complexes of Cu(II)GHK, we reacted it with two other thiols, 2-mercaptoethanol and sodium methanethiolate (NaSCH_3_). The main spectral changes triggered by the former reflected those observed for GSH; however, the d–d band disappearance was accompanied by the precipitation of an apparently insoluble Cu(I) species (Figure S22A). In the presence of NaSCH_3_, a 9 nm shift concomitant with a slight d–d band enhancement was observed initially, followed by the standard pattern of partial Cu(II) reduction to a Cu(I) species, followed by re-oxidation (Figure S22B,C). Noteworthy, in both cases, a characteristic band at 345 nm could be distinguished at the beginning of the reaction (Figure 4). These results indicate that all the three examined thiols formed transient ternary complexes with Cu(II)GHK via a sulfur atom, giving rise to a new absorption band at 345 nm, ascribed to S → Cu(II) LMCT,^55,56^ and the d–d band blue shift (Figure S23). A dynamic relationship between ternary Cu(II) complex formation and Cu(II) reduction depends on the reduction potential of the thiol in the order NaSCH_3_ < 2-mercaptoethanol < GSH. Notably, the formation of the thiolate ternary Cu(II) complex was efficient for monodentate thiols, not requiring the chelate ring formation described for cysteine complexes by Hanaki et al.^55,57,58^

Having confirmed the existence of the transient S–Cu(II)GHK species (S represents the monodentate thiolate coordination), we proceeded to establish its role in the Cu(II) reduction process. First, we employed NMR to check if GHK remained coordinated with Cu(I) after reduction. The reaction mixture, consisting of 2 mM GSH with 0.5 mM GHK and 0.45 mM Cu(NO_3_)_2_, was prepared in 50 mM sodium phosphate buffer, pH 7.4, under anaerobic conditions to ensure complete reduction of Cu(II) ions. The reaction was followed over 4 days in the NMR tube at 20 °C (Figure S24). The selected GHK signals were integrated and compared to the signal of the methyl group of acetate, which was a GHK counterion. They indicated that as long as oxygen-free conditions were present in the NMR tubes, the GHK apopeptide remained at the same level throughout the experiment. A control reaction without GHK was also performed under the same conditions (Figure S25). The separately recorded spectra of GSH, GSSG, and GHK in the absence of copper are presented in Figure S26 to aid the interpretation of the results. As shown in Figure S27, GSSG (determined directly) and Cu(II)GHK (inferred by the loss of GHK signals due to paramagnetic broadening) were the only chemical species at the end of the re-oxidation step. To better characterize the products, DOSY experiments were collected for the Cu(II)GHK reaction with GSH immediately after the GSH addition and after 22.5 and 93 h. These spectra are presented and analyzed in Figure S28. Two sets of diffusion parameters could be discerned. The one with the slower diffusion, at 1.3 × 10^–10^ m^2^·s^–1^, corresponded to a Cu(I)/GSH species and was absent at 93 h, while the other, at 3 × 10^–10^ m^2^·s^–1^, contained the GHK and GSSG apopeptide signals. The singlet of the acetate methyl group diffused faster, at 7 × 10^–10^ m^2^·s^–1^, as expected for a small non-interacting molecule. No signals from the GHK molecule bound to Cu(I) were seen. In particular, the His imidazole signals [the primary binding site expected for both Cu(I) and Cu(II)] exhibited single diffusion peaks aligned with other signals of the monomeric GHK apopeptide. Figure S29 presents the calculation of molecular volumes based on the DOSY signals of Glu β protons from the Cu(I)/GSH and GSSG spectra. The apparent volume of Cu(I)/GSH was 3 times larger than that of GSSG, corresponding well with the Cu(I)_4_GSH_6_ stoichiometry indicated by Fahrni et al.^47^ The above analysis clearly confirmed this complex as the only stable Cu(I) species throughout the reaction.

**Figure 5:**
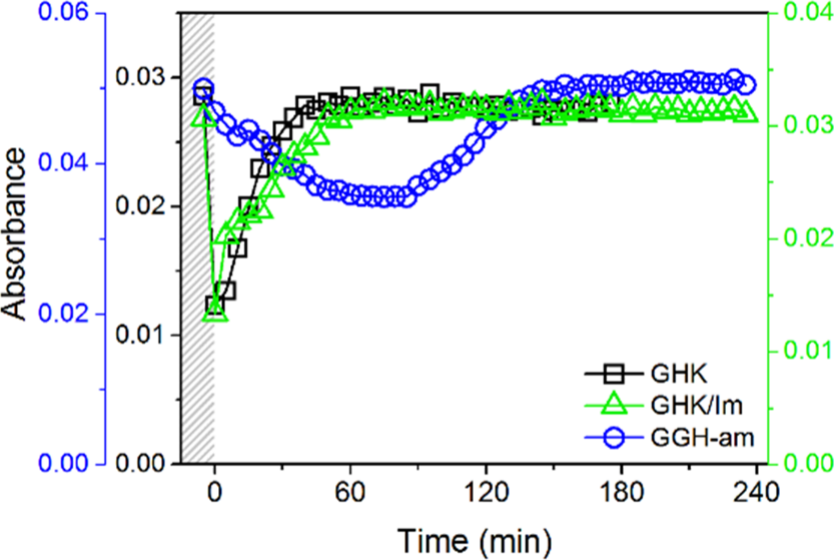
Absorbance changes of the d–d band triggered by GSH introduction into a solution of 0.50 mM GHK with 0.45 mM Cu(II) (606 nm, black squares), 50 mM imidazole (565 nm, green triangles), and 0.50 mM GGH-am with 0.45 mM Cu(II) (525 nm, blue circles).

The role of GSH-Cu(II)GHK in the redox process was further studied in the presence of a 100-fold excess of imidazole (Im) at 37 °C (Figure S30A). Im is able to displace water from the fourth equatorial site of Cu(II)GHK with a log ^c^*K* of 2.86 at pH 7.4.^1^ The calculated initial composition of Cu(II) species in this experiment, prior to GSH addition, was 96.6% Im-Cu(II)GHK and 3.4% Cu(II)GHK, confirmed by a blue shift of the d–d band maximum down to 565 nm, expected for the effective saturation of the fourth Cu(II) coordination site with Im. The addition of GSH to this ternary complex resulted in the partial Cu(II) reduction within the sample mixing time, exactly as in the absence of Im, followed by a similarly slow recovery of the Cu(II) species (Figure 5). No spectroscopic effects of the addition of GSH were noted in the d–d band region.

Since the ternary Im complex did not block the reduction of Cu(II) bound to GHK by GSH, control experiments for GGH amidated at the C-terminus were carried out. This peptide, together with its non-amidated counterpart, is the simplest ATCUN model, with amidation providing a better mimic of the peptide chain extension in ATCUN peptides/proteins.^31,59^ The GSH addition to Cu(II)GGH caused a partial Cu(II) reduction manifested by a decrease of the d–d band at 525 nm (Figure S30B), analogous to Cu(II)GHK and Im-Cu(II)GHK, but about 50-fold slower (Figure 5). In turn, the timespan of re-oxidation was similar for all the three systems, about 1 hour in our experimental conditions. There was no effect of GSH on the d–d band, as in Im-Cu(II)GHK. The features and course of Cu(II)GGH reaction with GSH were similar to other ATCUN systems studied in similar conditions.^42^

The impact of Im on the rate of Cu(II)GHK reduction was further studied at 20 °C. First, the rates recorded in the presence of varied Im concentrations were compared (Figure S31). A moderate ca. 2-fold drop of reduction rate was observed for 30 and 50 mM Im versus the sample without Im (see Table S3). Considering the near saturation of the fourth equatorial site in Cu(II)GHK at such Im excess, this suggested that GSH-Cu(II)GHK could not be a key species in the reduction. In another experiment, the reduction stage of the reaction without Im was followed at single wavelengths of 300, 345, and 606 nm, thereby shortening the dead time of signal detection to 10 s. The signals at 345 nm, characteristic for GSH-Cu(II)GHK, and at 606 nm reached their maximum within the shortened dead time (Figure S32). This was followed by an absorbance drop, reaching its minimum after 5 min and 10 min for CT and d–d band, respectively. The reduction progress monitored by d–d signal decay (representing all Cu(II) species) was in a perfect match with the absorbance changes at 300 nm (Figure S33).

A more precise analysis of these experiments required the knowledge of stability constant for GSH-Cu(II)GHK. It was determined on the basis of its CT band at 345 nm clearly recorded at low temperatures, where the reaction was sufficiently slow to enable the sequential collection of full UV–vis spectra. The data were obtained from a series of kinetic experiments where 0.45 mM Cu(II) and 0.5 mM GHK were reacted with 0.4–10 mM GSH (Figure S33). The sequentially recorded full UV–vis spectra revealed an isosbestic point at 320 nm in the initial reaction phase, indicating that Cu(I)_4_GSH_6_ (CT at 300 nm) was formed from GSH-Cu(II)GHK (CT at 345 nm) in a quantitative fashion in the given time window. This finding allowed us to calculate the absorption coefficients for GSH-Cu(II)GHK (ε_300_^II^,ε_345_^II^, Table S4) needed to determine its concentration along with the reaction progress. Details are described in the Supporting Information section “*Calculation of the GSH-Cu(II)GHK stability constant*”. The concentrations of all relevant complex species and the conditional stability constant ^C^*K*_7.4_ for GSH-Cu(II)GHK (eq 1) were obtained from extrapolation of *A*_300_ and *A*_345_ to the zero time point for each reaction using an empiric first-order reaction equation (Table S5). This was necessary to make valid assumptions about the concentration of GSH, which was consumed during the reaction in a non-linear fashion.
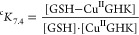
1

The final value of log ^c^*K*_7.4_, averaged over the four lowest GSH concentrations, was 2.91 ± 0.06. The limitation of this data range for calculations was dictated by the lowest extent of side reactions such as GSSG production, which influenced the absorbance intensities in the CT region.

The ^C^*K*_7.4_ for GSH-Cu(II)GHK enabled us to calculate the initial compositions of reaction solutions, immediately after the GSH additions. Such calculations are provided in Table S6 for the kinetic experiments in the absence and presence of Im. The ^C^*K*_7.4_ values for GSH-Cu(II)GHK and Im-Cu(II)GHK are identical within the experimental error,^1^ confirming that a 30- and 50-fold excess of Im over GSH diminished the ternary complex share to single percentage points versus its estimated initial presence at 40% of Cu(II) in the absence of Im. These findings indicated that the ternary GSH-Cu(II)GHK complex was not necessary for the Cu(II) reduction but participated in this process in the absence of alternative ternary ligands.

The quantitative redox aspects of the examined system were investigated by cyclic voltammetry (CV). The representative cyclic curves of Cu(II)GHK are presented in Figure S34A. The main electrochemical feature of the binary complex is an irreversible metal ion reduction at around −0.60 V (vs AgCl/Ag), followed by a redox center oxidation near −0.1 V. A significant shift in the position of the anodic response relative to the one expected upon the assumption of one-electron transfer (i.e., Δ*E* is higher than 60 mV) suggests that a considerable structural change occurs in the copper coordination sphere upon reduction.^27,60,61^ The reorganized structure of the complex does not seem to meet the stereochemical requirements of the Cu(I) cation, as evidenced by the shift of anodic response along with successive scans, accompanied by a peak shape alteration alongside the scan rate decrease (Figure S34A, inset). A likely explanation is an exclusion of the amide nitrogen from the coordination sphere upon reduction, as Cu(I) is unable to deprotonate and coordinate it.^62^ This leads to the release of free Cu(I) ions, which tends to adsorb and accumulate on the electrode surface during the redox cycling. This feature corresponds to the absence of Cu(I)-GHK species demonstrated by NMR. Irreversible oxidation of Cu(II)GHK to a Cu(II)^I^ species was seen at 1.3 V.^27,63^ A high potential value makes this process irrelevant for the studied reaction.

**Figure 6:**
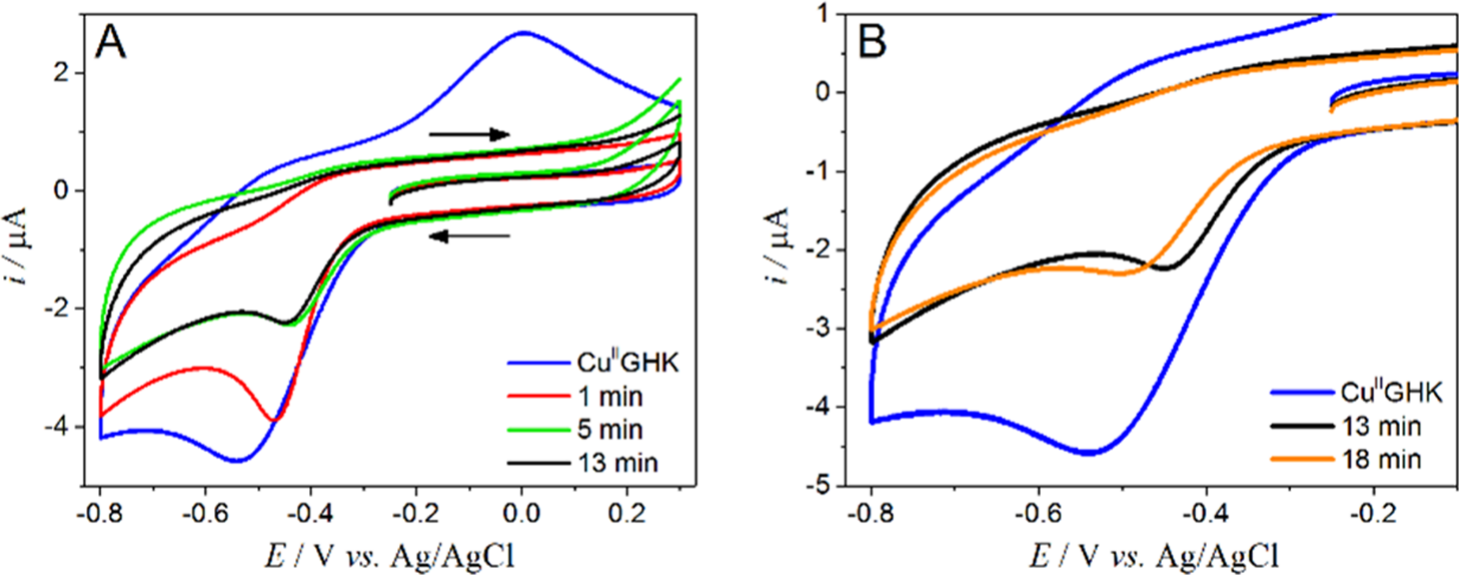
Selected CV curves recorded for 0.50 mM GHK with 0.45 mM Cu(II) in the presence of 1.0 mM GSH in 100 mM KNO_3_; υ = 100 mV/s; panel (A) depicts current responses for the Cu(II)GHK binary complex (blue line) and three first measurements after GSH addition (red, green, and black line, respectively); panel (B) in addition to the voltammogram of Cu(II)GHK shows the last curve from panel (A) and the one representing steady state; pH = 7.4, room temperature.

Tracing the course of the Cu(II)GHK reaction with GSH by electrochemical methods is a difficult task since the thiol group alone undergoes a series of oxidation reactions in the range of positive potentials.^64^ The situation gets even more complicated when an interaction with copper is examined due to multiple interconnected chemical and electrochemical redox events, yielding a continuous change of electrode signals. Therefore, we focused on the range of negative potentials, where only the processes specific to the Cu(II)GHK system occurred (Figure 6). No reduction signal was detected in the above potential window in the GHK absence under anaerobic conditions enabled by an argon blanket; hence, the observed electrochemical effects originated in the GHK complex (Figure S34B). The GSH addition influenced both the position and the current of the cathodic peak. First, a dynamic cathodic current drop along with the signal shift toward less negative potentials was observed (Figure 6A), indicating that coordination changes caused by GSH favored the electron transfer. The Cu(I) species formed electrochemically was rapidly captured by GSH, causing an immediate disappearance of the follow-up anodic peak (ca. −0.1 V). After reaching the minimum value of the current, the reduction peak shifted back to negative values and remained unaffected unless the oxygen access was provided (Figure 6B). This profile of electrochemical changes is consistent with the spectroscopic results discussed above. Since the cathodic signal shift toward less negative potentials was observed previously as a consequence of ternary complex formation by 3N species,^65^ such temporary fluctuations in the reduction peak position along with a significant current decrease fit well with the transient GSH-Cu(II)GHK species. A longer-term signal stabilization at a potential slightly higher than for Cu(II)GHK correlates with the accumulation of GHK-Cu(II)GHK, accompanying the loss of Cu(II) due to reduction, as explained above.

The above results allowed us to propose the scheme of Cu(II)GHK interaction with GSH. Reaction 2 provides the overall stoichiometry of Cu(II) reduction, where [CuGHK]* is a postulated reduction intermediate detected by electrochemistry but not visible to spectroscopic methods due to its very short lifetime, with the follow-up rapid formation of Cu(I)_4_GSH_6_ driven by a huge difference of Cu(I) affinities to nitrogen ligands (nano- to picomolar)^66^ and GSH (attomolar).^47^
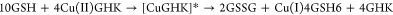
2

However, the corresponding oxidation of GSH to GSSG was suprastoichiometric, as indicated by the correlation of the extent of Cu(II) reduction with the initial GSH concentration (see Figure S33). A complete reduction was achieved for 2 mM GSH under aerobic conditions, but measurements under near-anaerobic conditions (0.5–1% O_2_) clearly indicate a prominent impact of oxygen already in the reduction phase. For 1 mM GSH, the reduction endpoint calculated from the minimum of d–d absorption band was ca. 60% for aerobic and 75% for low oxygen conditions (Figure S9). Comparing the pace of this process with a low oxidation susceptibility of control GSH solutions, one can conclude that the additional GSH oxidation was catalyzed by Cu(II)/GHK complexes rather than the free Cu^2+^ ion, limited to less than picomolar concentrations by very strong chelation by GHK. This observation is corroborated by electrochemistry, which demonstrated the redox capability of Cu(II)/GHK complexes. It is also in agreement with the findings presented by Compton et al.^67^

The parallel GSH oxidation mechanism established in the literature includes the electron transfer from Cu(I) to GSH by a transient superoxide anion-radical, which yields the thiyl radical, finally recombining into GSSG, according to the following reactions^45,68,69^
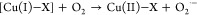
3
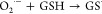
4
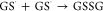
5where [Cu(I)–X] represents all intermediate Cu(I) species prone to an oxygen-induced oxidation process.

**Scheme 1:**
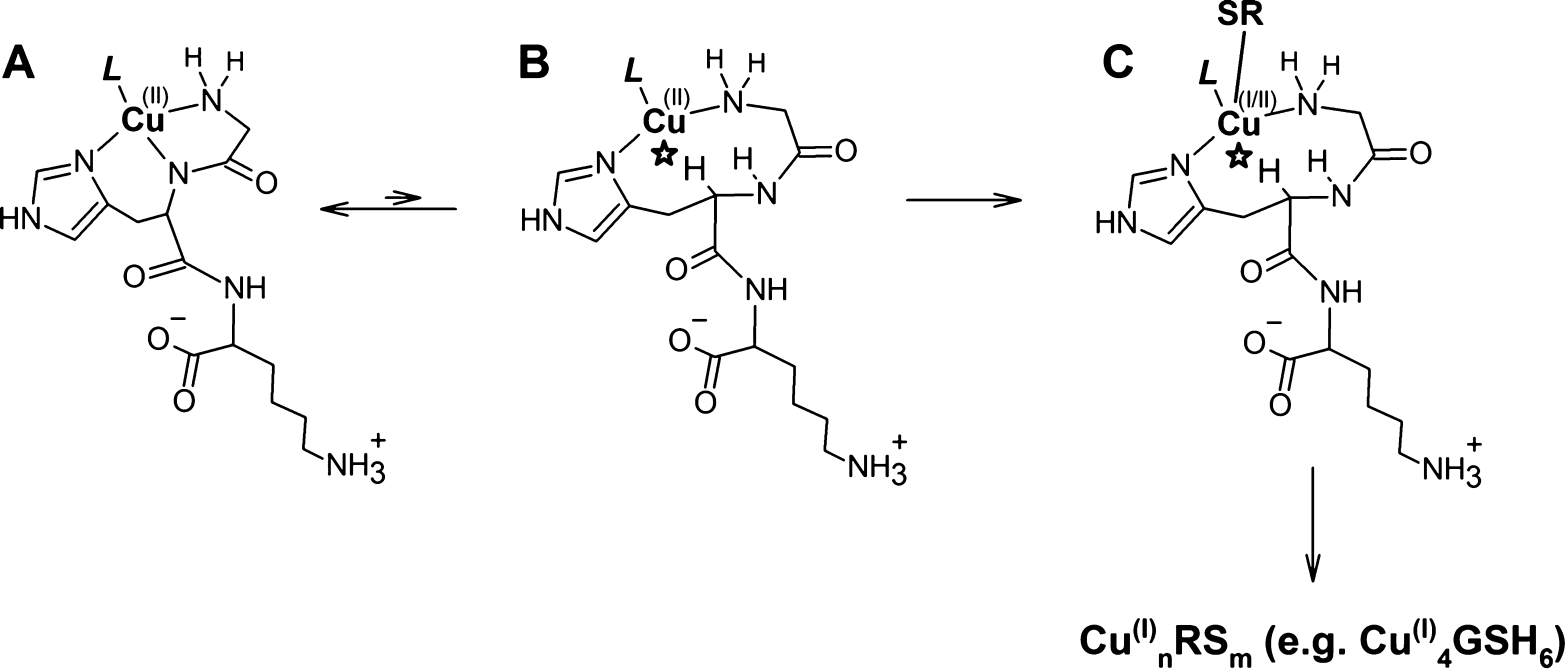
Proposed Structural Scheme of the Cu(II) Reduction Reaction at pH 7.4 in an Aqueous Solution The initial L-Cu(II)GHK complex (L = H_2_O, a thiolate including GSH or Im) (A) spontaneously rearranges to a minor reactive intermediate [Cu(II)GHK]* by the peptide nitrogen detachment and reprotonation (B), where the steric hindrance from the peptide chain (*) makes it a T-shaped three-coordinate transient susceptible to the “axial” approach by the thiolate reductant enabling the inner sphere electron transfer (C).

What remains to be established is the nature of the transient [CuGHK]* species in reaction 2 and the pathway of its formation. A plausible mechanism can be inferred from the recent finding that the redox activity of 4N species of GGH and other ATCUN complexes^70,71^ involved a 2N kinetic intermediate, providing a quasi-reversible Cu(II)/Cu(I) redox pair. An analogous 2N species with a N_im_ + NH_2_ donor set was also observed in stopped-flow studies of Cu(II) binding to the GHTD-am peptide, which shares the Cu(II) coordination mode with GHK.^72^ This structure is inferred for [Cu(II)GHK]*, as presented in Scheme 1. The peptide nitrogen provides effective stabilization of higher copper oxidation states [Cu(II) over Cu(I) and Cu(III) over Cu(II)] by electrostatic interactions.^63,73,74^ Therefore, its exclusion from the coordination sphere in [Cu(II)GHK]* enables its susceptibility to reduction by GSH according to reaction 6.
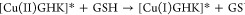
6

Furthermore, a steric hindrance from the GHK peptide chain may actually exclude a water molecule from the vacated equatorial position, making this complex three-coordinate in a roughly T-shaped ligand arrangement. This hypothetical steric hindrance is marked with a star in Scheme 1. It should be noted that [Cu(II)GHK]* is actually a generic term comprising the ligand L, which may be a water molecule, an equatorially coordinated GSH, or another ternary partner, such as Im.

Such species can be universally approached by an “axial” GSH or other thiol, as illustrated in Scheme 1. This proposal readily explains the merely ca. 2-fold slowdown of the Cu(II) reduction reaction in the Im ternary complex. For L being the non-thiol, there is only one (“axial”) pathway of the inner sphere electron delivery to Cu(II) by the reducing thiolate, while for L being GSH or other thiol, there are two possible ways, the “axial” and the “equatorial” ones. The respective reduction rates are additionally modulated by the nucleophilic properties of ternary ligands L, which is indicated by electrochemical measurements, and by thermodynamic stability of [Cu(I)GHK]* with various L. The inner sphere electron transfer from the thiol, demonstrated for similar systems by Holwerda et al.,^75^ is followed by detachment of GHK assisted by an additional thiol molecule—toward the final Cu(I)/thiolate structure, for example, Cu_4_GSH_6_.

Under near-anaerobic conditions, the Cu(II) reduction progressed until GSH was exhausted by oxidation to GSSG and tight complexation of generated Cu(I). Then, a steady-state phase followed, co-habitated by Cu(I) and Cu(II) complexes. This indicates that GSH in the Cu(I)_4_GSH_6_ complex is not able to reduce the external Cu(II) ions. This phase was terminated by allowing the access of dioxygen. The Cu(I) re-oxidation to Cu(II) driven by it also has a radical reaction mechanism. The similarity of its rate in the presence of various Cu(II)-chelating His peptides and the absence of any covalent oxidation products except GSSG indicated that Cu(I) was oxidized within the Cu(I)_4_GSH_6_ complex, followed by rapid Cu(II) capture by His peptides. In their absence, Cu(II)GSSG and GSSG were the sole final reaction products.^42^ The re-oxidation mechanism is described by reactions 3–5 presented above.^45,68,69^

The relevance of GSH-Cu(II)GHK strongly depends on the biological compartment, limited by its relatively low stability constant but offset by its relatively long lifetime. Bearing in mind the extracellular presence of GHK and the absence of covalent reaction side products, we imply that this complex may be a missing link in the process of clean delivery of Cu(I) ions for transmembrane transporters of the CTR family because extraneous radical species are efficiently scavenged by GSH as in reactions 4 and 5. Other Cu(II) complexes in blood, such ATCUN motifs present, for example, in HSA, or amino acid complexes, for example, Cu(II)His_2_, are virtually impossible to reduce in blood serum conditions. Nevertheless, we tend to advocate a small-molecule Cu(I) delivery system as more compatible with the large extracellular domain of human CTR1, as opposed to putative cell surface Cu(II) reductases, which are known in yeast whose CTR1 has only a very small extracellular part.^77^ This idea is further supported by the ability of ternary ligands, both imidazoles and thiols, to tune its rate. Analogous species could also form and act intracellularly, given the abundance of XH motifs in cellular proteins,^78^ and the known strict homology of various XH motifs in terms of Cu(II) coordination properties.^49−52^

## Conclusions

In the present work, we reported data on the glutathione-mediated reduction of Cu(II) bound to GHK and GGH peptides, followed by spectroscopic methods (UV–vis, CD, EPR, and NMR) and cyclic voltammetry. Our comprehensive study sheds new light on the biological role of the widely studied Cu(II)GHK complex, revealing it as a good candidate for extracellular and intracellular Cu(I) supply via ternary complexes with thiol compounds, such as GSH.

## Supporting Information Available

Experimental details, additional spectroscopic and voltammetric experiments, mass spectrometry data, and detailed description of performed calculations (PDF)
