# Supplementary material for: Intermediate Cu(II)-Thiolate Species in the Reduction of Cu(II)GHK by Glutathione: A Handy Chelate for Biological Cu(II) Reduction
Source: Inorg Chem. 2021 Nov 15;60(23):18048–57. doi: 10.1021/acs.inorgchem.1c02669 (PMC8653159; doi:10.1021/acs.inorgchem.1c02669)
Supplement: Supplementary file 1 — ic1c02669_si_001.pdf [file ic1c02669_si_001.pdf]

Supporting Information

Intermediate Cu(II)-thiolate species in reduction of Cu(II)GHK by glutathione: a handy chelate for biological Cu(II) reduction

Iwona Ufnalska,[a] Simon C. Drew,[c] Igor Zhukov,[b] Kosma Szutkowski,[d] Urszula E. Wawrzyniak,[a] Wojciech Wróblewski,[a] Tomasz Frączyk,*[b] Wojciech Bal,*[b]

[a] Chair of Medical Biotechnology, Faculty of Chemistry, Warsaw University of Technology, Noakowskiego 3, 00-664 Warsaw, Poland.

[b] Institute of Biochemistry and Biophysics, Polish Academy of Sciences, Pawińskiego 5a, 02-106 Warsaw, Poland

[c] Department of Medicine (Royal Melbourne Hospital), The University of Melbourne, Victoria, 3010, Australia

[d] NanoBioMedical Centre, Adam Mickiewicz University, Wszechnicy Piastowskiej 3, 61-614 Poznań, Poland

Corresponding Authors E-mails:

* wbal@ibb.waw.pl, tfraczyk@ibb.waw.pl

Table of Content

Nie znaleziono żadnych pozycji spisu treści.

Materials and methods

Chemicals and reagents

The Gly-His-Lys, copper(II) nitrate hydrate, potassium nitrate, sodium dihydrogen phosphate, sodium hydrogen phosphate, HEPES, nitric acid, hydrogen chloride, sodium hydroxide, and potassium hydroxide (used for pH adjustment) were purchased from Sigma-Aldrich and were used without additional purification. The Gly-Gly-His-am (amidated at the C-terminus) was synthesized using standard Fmoc/tBu procedure with the use of the automated microwave peptide synthesizer Liberty (CEM). Cleavage reaction was carried out using cocktail composed of: TFA/TIS/water 95:2.5:2.5 (v:v:v) for two hours. Afterward, the peptide was precipitated from the cleavage solution with cold diethyl ether. After centrifugation, the peptide was dissolved in water and lyophilized. The peptide was purified by HPLC (Waters) with UV detection set at 220 nm. All solutions were prepared using deionized water (Millipore). The identity and purity of the peptide were checked on ESI-MS (Waters).

UV-Vis and CD

UV-Vis spectra were recorded on the Lambda 950 UV/VIS/NIR spectrophotometer (PerkinElmer), and CD spectra on the J-815 spectropolarimeter (Jasco), both equipped with cuvette temperature control systems. Time-wise measurements of the samples containing 0.50 mM GHK or GGH, 0.45 mM $Cu(NO_3)_2$, and 0.4-10.0 mM GSH, pH 7.4, were carried out over the spectral range of 230–850 nm (UV-Vis) and 280–800 nm (CD). The reaction course was followed at different temperatures, ranging from 37°C to 5-7°C (denoted as 5°C in the main text), which was the lowest technically available temperature. The optical path length was 1 cm in all cases.

Calculation of the GSH-Cu(II)GHK stability constant

The quantitative analysis performed for a reaction of 0.50 mM GHK and 0.45 mM Cu(II) with 1.0 mM GSH mM at 5°C consisted of:

(1) Determination of absorption coefficients at 300 nm and 345 nm

a. ε_{300}^{I}, ε_{345}^{I} (absorption coefficients for $Cu(I)_4GSH_6$)

These absorption coefficients were acquired from control reaction between GSH and $Cu(NO_3)_2$, in the absence of GHK, where $Cu(I)_4GSH_6$ and Cu(II)GSSG were the only copper-containing species with a significant absorption above 250 nm (Figure S1). First, ε_{625} for Cu(II)GSSG was determined from the last recorded spectrum, assuming that all Cu(II) was bound to GSSG at the end of the re-oxidation reaction**.** The obtained ε_{625} value, 62 $dm^3 \cdot mol^{-1} \cdot cm^{-1}$, is in good agreement with the literature data (60 $dm^3 \cdot mol^{-1} \cdot cm^{-1}$).[1] This allowed us to calculate the Cu(II) concentration for any given time point of the reaction using ε_{625} and A_{625} ($Cu(I)_4GSH_6$ does not absorb in the visible range). This value yielded the Cu(I) concentration from the mass balance. However, spectral intensities had to be corrected by taking into account the differences between the times at which the data were recorded at given wavelengths during the sweep of the spectrum from high to low wavelengths; $\Delta t_1 = t_{625} - t_{345}$ and $\Delta t_2 = t_{625} - t_{300}$ were noted. As the $Cu(I)_4GSH_6$ re-oxidation to Cu(II)GSSG occurs on a minutes timescale, the linear approximation of the reaction course between the first two measurement points ($A_{625}(t)$) was found to be sufficiently accurate. As a result, time-corrected absorption values at 625 nm ($A_{625}(t_{345})$ and $A_{625}(t_{300})$), and hence the corresponding Cu(II) and Cu(I) concentrations at those time points were obtained. Finally, ε_{300}^{I} and ε_{345}^{I} were determined from the same spectra using A_{300} and A_{345}, respectively (see Table S4).

b. ε_{300}^{II}, ε_{345}^{II} (absorption coefficients for GSH-Cu(II)GHK)

The absorption values at 345 and 300 nm for GSH-Cu(II)GHK could not be used in calculations as read directly from spectra due to significant overlap with the bands of $Cu(I)_4GSH_6$ and to a lesser extent with those of Cu(II)GHK and GHK-Cu(II)GHK (Figure S1, dashed line). In order to circumvent this problem, we resorted to the fact that no species other than GSH-Cu(II)GHK absorbed significantly in the 380-450 nm range. The spectra were transformed into the wavenumber domain to obtain a symmetrical Gaussian absorption band centered at 28 980 cm^{-1} (345 nm). The symmetry was confirmed for the analogous band formed in the presence of $NaSCH_3$, where the CH_3S-Cu(II)GHK ternary complex was the sole spectroscopic species at the beginning of the reaction (Figure 3, inset). In this way, we could determine A_{300} for GSH-Cu(II)GHK from its symmetrical Gaussian low-energy counterpart, A_{406}. Then equations S1 and S2

$$A_{406} = \varepsilon_{300}^{II} \cdot c \cdot l \qquad \text{(S1)}$$

$$A_{345} = \varepsilon_{345}^{II} \cdot c \cdot l \qquad \text{(S2)}$$

transformed into equation S3

$$\frac{A_{406}}{A_{345}} = \frac{\varepsilon_{300}^{II}}{\varepsilon_{345}^{II}} \qquad \text{(S3)}$$

gave the final expression for ε_{300}^{II} (S4):

$$\varepsilon_{300}^{II} = \frac{A_{406}}{A_{345}} \cdot \varepsilon_{345}^{II} \qquad \text{(S4)}$$

These calculations were performed for the initial spectra of the GSH titration (with the Cu(II)GHK spectrum subtracted for the reasons stated above). A satisfactory spread of the values was obtained, except of 10 mM GSH, giving an average value of 0.082, as shown in Table S1.

The ε_{345}^{II} value calculations were based on the assumption of direct and sole formation of $Cu(I)_4GSH_6$ (manifested at 300 nm) from GSH-Cu(II)GHK (manifested at 345 nm) over a given time window. It was justified by the presence of the isosbestic point at around 320 nm. In other words, in this time window, the increase of [$Cu(I)_4GSH_6$] multiplied by 4 to adjust for the stoichiometry ($\Delta c_{Cu(I)_4^IGSH_6}$) was equal to the decrease of [GSH-Cu(II)GHK] ($\Delta c_{GSH-Cu^{II}GHK}$). Therefore, respective absorbance changes (ΔA_{300}, ΔA_{345}) could be written as follows:

$$\Delta A_{300} = \varepsilon_{300}^{I} \cdot \Delta c \cdot l - \varepsilon_{300}^{II} \cdot \Delta c \cdot l \qquad \text{(S5)}$$

$$\Delta A_{345} = \varepsilon_{345}^{II} \cdot \Delta c \cdot l - \varepsilon_{345}^{I} \cdot \Delta c \cdot l \qquad \text{(S6)}$$

where $\Delta c = 4 \cdot |\Delta c_{Cu_4^IGSH_6}| = |\Delta c_{GSH-Cu^{II}GHK}|$.

The equations S5 and S6 can be presented in the following simplified form (S7):

$$\frac{\Delta A_{300}}{\Delta A_{345}} = \frac{\varepsilon_{300}^{I} - \varepsilon_{300}^{II}}{\varepsilon_{345}^{II} - \varepsilon_{345}^{I}} \qquad \text{(S7)}$$

from which ε_{345}^{II} value was calculated.

The ΔA_{300} value was also time-corrected with regard to the time point of the A_{345} measurement using the equation obtained to extrapolate the data for the reduction process, as described below.

(2) Extrapolation to zero time point (A_{300}^0 and A_{345}^0)

A_{300}^0 and A_{345}^0 values were acquired by individual extrapolations at given wavelengths using the appropriate 1st order kinetic equation fitted to four initial reaction time points, separately for each GSH concentration (Table S5). The R^2 was 0.9992 ± 0.0017 for A_{300} and 0.9974 ± 0.0080 for A_{345}.

The apparent paradox of zero-time presence of $Cu(I)_4GSH_6$ is justified by a very fast initial rate of Cu(II) reduction; see main text for details of reaction course and mechanism.

(3) Determination of [$Cu(I)_4GSH_6$] and [GSH-Cu(II)GHK] at 0 s and stability constant for the GSH-Cu(II)GHK complex

Based on the data obtained in previous steps, the concentrations of both complexes were calculated for each GSH concentration separately according to the following set of equations:

$$A_{300}^0 = c_{Cu^I}^0 \cdot \varepsilon_{300}^{I} + c_{Cu^{II}}^0 \cdot \varepsilon_{300}^{II} \qquad \text{(S8)}$$

$$A_{345}^0 = c_{Cu^I}^0 \cdot \varepsilon_{345}^{I} + c_{Cu^{II}}^0 \cdot \varepsilon_{345}^{II} \qquad \text{(S9)}$$

results of which are listed in Table S5.

The calculated values were applied to obtain the GSH-Cu(II)GHK ternary stability constant (S11) from the reaction (S10):

$$\text{GSH + Cu(II)GHK} \rightleftharpoons \text{GSH-Cu(II)GHK} \quad \text{(S10)}$$

$$K_T = \frac{[\mathrm{GSH{-}Cu^{II}GHK}]}{[\mathrm{GSH}]\cdot[\mathrm{Cu^{II}GHK}]} \quad \text{(S11)}$$

In these calculations, the minor contribution of GHK-Cu(II)GHK was ignored, as speciation calculations indicated that its contribution never exceeded 1.4% of total copper in the reaction. The residual GSH concentration was calculated assuming 1 GSH molecule being consumed per one GSH-Cu(II)GHK complex and 1.5 GSH equivalent being engaged into one Cu(I) ion coordination (in accord to $Cu(I)_4GSH_6$). Cu(II)GHK concentration was determined as a difference between total Cu (0.45 mM) and calculated concentrations of GSH-Cu(II)GHK and Cu(I) (Table S5).

EPR

The EPR spectra were acquired at 77K using a CMS8400 X-band (9.42 GHz) spectrometer (Adani, Belarus) fitted with a TE102 cavity and a quartz cold finger insert (Wilmad, WG-816-B-Q). Measurements were made using the following settings: microwave frequency, 9.42 GHz; microwave attenuation, 10 dB; magnetic field sweep rate, 8 gauss s^{-1}; magnetic field modulation amplitude, 8 gauss; magnetic field modulation frequency, 100 kHz; field resolution, 4096 points; receiver gain, 100; time constant, 100 ms; averages, 10. Baseline correction of spectra was carried out by subtracting the spectrum of 10% glycerol in water. Normalization of the microwave absorption to unit area was carried out by double-integration of the experimental first-harmonic spectra.

NMR

The NMR experiments were performed at 298 K utilizing a Varian Inova 500 NMR spectrometer operating at 11.7 T magnetic field (^{1}H resonance frequency 500.606 MHz) equipped with three channels, *z*-gradient unit Performa IV, and ^{1}H/^{13}C/^{15}N triple resonance probehead with inverse detection. The NMR sample contained initially 2 mM GSH and 0.5 mM GHK dissolved in 90%/10% H_2O/D_2O, 50 mM sodium phosphate buffer, at pH 7.4 to which 0.45 mM $Cu(NO_3)_2$ was added, followed by a pH check. The control sample, without GHK, was prepared under the same conditions. Additional control samples contained pure 1 mM GHK, GSH and GSSG dissolved in the same buffer. The samples were measured in a side-by-side regime. Assignments of the ^{1}H and ^{13}C resonances were based on a joint analysis of one-dimensional ^{1}H spectra, two-dimensional ^{1}H-^{1}H TOCSY (80 ms mixing time), and ^{1}H-^{13}C HSQC recorded on the natural abundance of the ^{13}C isotope. All spectra were referenced indirectly with respect to external sodium 2,2-dimethyl-2-silapentane- 5-sulfonate (DSS) using Ξ = 0.251449530 ratio for the ^{13}C dimension.[2]

Translation diffusion coefficients (D_{tr}) were measured with Pulsed Field Gradient Spin Echo NMR (PGSE NMR) experiment at 298 K. The values of D_{tr} were independently obtained for two signals at 3 and 3.15 ppm. The raw PGSE data was initially manipulated using MestReNova software to correct phase shifts as well as base-line using the Bernstein Polynomial Fit method. Next, extracted spin echo signal decays at 3 and 3.15 ppm were linearized vs G^2 and diffusion coefficients were obtained from the slope of the linear fit using Origin 2021 software. The data points were fit to the general Stejskal-Tanner equation (S12):[3]

$$I = I_0\, exp(-D(G\gamma\delta)^2(\Delta - \delta/3)), \quad \text{(S12)}$$

where γ is the ^{1}H gyromagnetic ratio, Δ is diffusion time [s], *G* is the magnetic field gradient strength [T/m], and δ is gradient duration [s]. The experimental data were acquired in 25 gradients steps, using diffusion time 50 ms, and gradient duration was set typically to 2 ms. The DOSY spectra and D_{tr} values were also calculated using the Bayesian method[4] embedded in MestReNova software 12.04.

Electrochemistry

Electrochemical measurements were performed using the CHI 1030 potentiostat (CH Instrument, Austin, USA) in a three-electrode arrangement: Ag/AgCl electrode as a reference electrode, platinum wire as an auxiliary electrode, and glassy carbon electrode (GCE, BASi, 3 mm diameter) as a working electrode. Prior to each measurement, the GCE surface was carefully polished to a mirror-like surface with the use of alumina suspensions (1.0 and 0.3 μm in succession) on a Buehler polishing cloth. To remove residual abrasive particles, the working electrode was sonicated for 1 min and then rinsed thoroughly with distilled water. The conductivity of the solution was provided by 100 mM KNO_3, therefore strict control of pH was demanded (small aliquots of concentrated KOH or HNO_3 solutions were used, if required). To avoid the undesired influence of dissolved oxygen and free copper ions, all electrochemical experiments were carried out under argon with an excess of the ligand (peptide-to-copper(II) ratio was 1.0:0.9). Cyclic voltammetry (CV) curves at different scan rates and different potential ranges were recorded.

Supplementary Tables

Table S1 Results of ε_{300}^{II} calculation.

**GSH / mM**	0.4	0.8	1.0	1.5	2.0	3.0	5.0	7.5	10.0
$\mathbf{A_{406}/A_{345}}$	0.083	0.082	0.089	0.0830	0.077	0.082	0.078	0.083	0.062
$\overline{\mathbf{A_{406}/A_{345}}}$	0.082								

Table S2. Spin Hamiltonian parameters used to simulate the EPR spectra in Figure S18 and comparison with previous studies at room temperature.

	**g^**	$\mathbf{g_{\|\|}}$	**A^(^{65}Cu)**	$\mathbf{A_{\|\|}}$**(^{65}Cu)**	$\mathbf{A_{iso}^{N1}}$	$\mathbf{A_{iso}^{N2}}$	$\mathbf{A_{iso}^{N3}}$	$\mathbf{A_{iso}^{N4}}$
**Cu(II)GHK**								
77K	2.051	2.233	15.4	202.0	10.9	12.3	14.3	n.a.
Room temperature[5]	2.056	2.229	15.3	198.3	10.7	12.2	14.0	n.a.
**GHK-Cu(II)GHK**								
77K	2.045	2.212	23.6	212.0	11.2	13.6	14.0	14.3
Room temperature[5]	2.057	2.192	22.7	206.2	11.2	13.6	14.0	14.3
**GSH-Cu(II)GHK**	2.038	2.175	34.0	206.1	n.d.	n.d.	n.d.	n.a.

n.d. = not determined
n.a. = not applicable

Table S3. The reaction rate constants acquired using 1st order equation fitted to the initial 900-1000 s of the curves presented in Fig. S29.

c_{Im} / mM	0	10	30	50
k / s^{-1}	0.00932	0.00687	0.00437	0.00494

Table S4. Values of calculated molar absorption coefficients, further used for GSH-Cu(II)GHK stability constant determination.

Cu(II)GSSG	GSH-Cu(II)GHK		$Cu(I)_4GSH_6$	
ε_{625}	ε_{345}^{II}	ε_{300}^{II}	ε_{300}^{I}	ε_{345}^{I}
$dm^3 \cdot mol^{-1} \cdot cm^{-1}$				
62	3 145	258	8 672 (2 168 per Cu(I))	2664 (666 per Cu(I))

Table S5. Results of extrapolation to zero time point and corresponding $logK_{7.4}$ values for GSH-Cu(II)GHK complex.

C_{GSH} / mM	A_{300}^{0}	A_{345}^{0}	$c_{Cu^{I}}$ / mM	$c_{GSH-Cu^{II}GHK}$ / mM	$logK_{7.4}$
0.4	0.074	0.291	0.024	0.087	2.969
0.8	0.177	0.420	0.068	0.119	2.893
1.0	0.254	0.470	0.102	0.128	2.907
1.5	0.373	0.523	0.156	0.133	2.866
2.0	0.480	0.613	0.203	0.152	3.016
3.0	0.561	0.675	0.239	0.164	3.150
5.0	0.673	0.723	0.290	0.168	< 0
7.5	0.675	0.732	0.291	0.171	< 0
10.0	0.664	0.879	0.280	0.220	< 0

Table S6 The contribution of individual species provided for 0.50 mM GHK with 0.45 mM Cu(II) in the presence of 1.0 mM GSH without/with different concentrations of imidazole at pH 7.4; the values in table are molar fractions of Cu(II) expressed as a percentage; stability constant values used for calculations were acquired from Bossak-Ahmad et al.[5]

C_{Im} / mM	Cu(II) / %	Cu(II)GHK / %	GHK-Cu(II)GHK / %	Im-Cu(II)GHK / %	GSH-Cu(II)GHK / %
0	0.00	59.29	1.40	0.00	39.36
10	0.00	13.66	0.36	75.47	10.52
30	0.00	5.34	0.14	90.33	4.23
50	0.00	3.32	0.09	94.01	2.65

Supplementary Figures

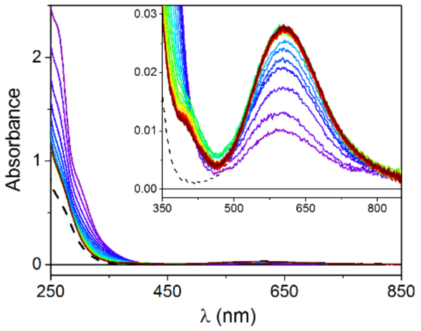

Figure S1. UV-Vis spectra collected with 5 min intervals for 0.50 mM GHK with 0.45 mM Cu(II) in the presence of 1.0 mM GSH in HEPES buffer at pH=7.4, T=37°C.

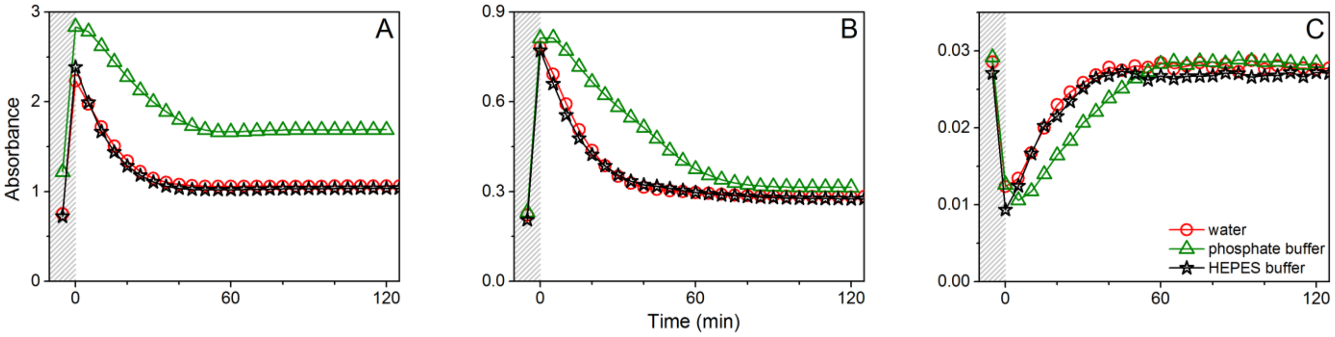

Figure S2. Absorbance changes at (A) 255 nm, (B) 300 nm, and (C) 606 nm for 0.50 mM GHK with 0.45 mM Cu(II) in the presence of 1.0 mM GSH in different media: water (red circles), 50 mM phosphate buffer (green triangles) and 50 mM HEPES buffer (black stars); pH=7.4, T=37°C; absorbance values for the binary complex are presented in the shaded field.

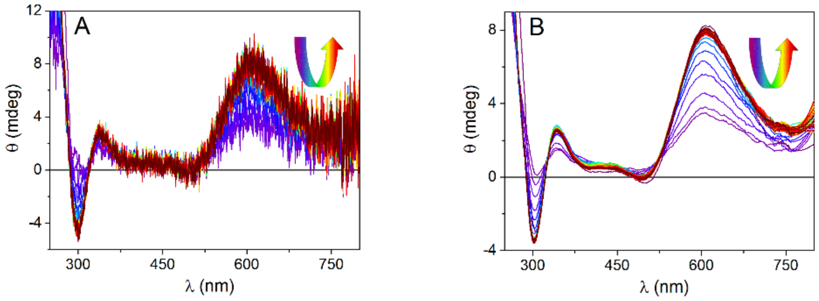

Figure S3 (A) raw CD spectra collected with 5 min intervals for 0.50 mM GHK with 0.45 mM Cu(II) in the presence of 1.0 mM GSH at pH=7.4, T=37°C; the curved arrow indicates the rapid loss followed by the slow recovery of *d-d* band; panel (B) depicts results of spectra smoothing with Savitzky-Golay method (points of window: 75, polynomial order: 2).

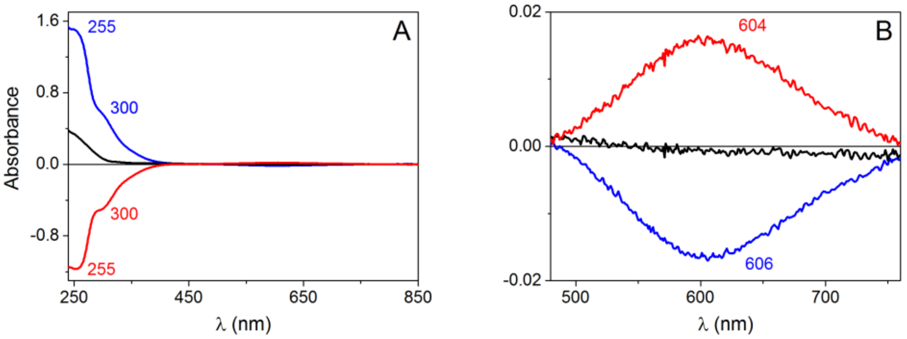

Figure S4. Differential absorption spectra calculated for 0.50 mM GHK with 0.45 mM Cu(II) in the presence of 1.0 mM GSH at different time points: the blue line represents changes that occurred within the mixing time (expressed as a difference between the first spectrum recorded after GSH introduction and Cu(II)GHK spectrum), the black line represents overall changes that took place during the reaction (expressed as a difference between the last spectrum recorded after GSH introduction and Cu(II)GHK spectrum), the red line represents changes that took place during Cu(II)GHK complex recovery step (expressed as a difference between the spectrum recorded after 40 min and immediately after GSH introduction); pH=7.4, T=37°C.

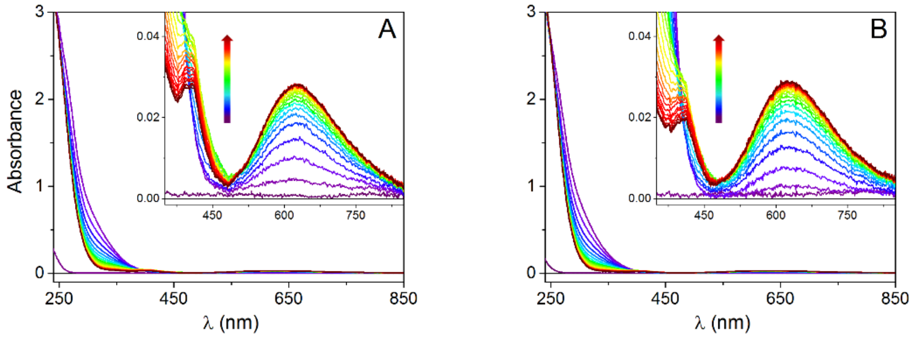

Figure S5. UV-Vis spectra collected with 5 min intervals for 1.0 mM GSH with 0.45 mM Cu(II) in (A) phosphate buffer and (B) HEPES buffer at pH=7.4, T=37°C; the control in pure water was not provided for technical reasons - GSH reaction with copper(II) is fast, whereas pH adjustment in water is a time-consuming task, which would result in the loss of the first points.

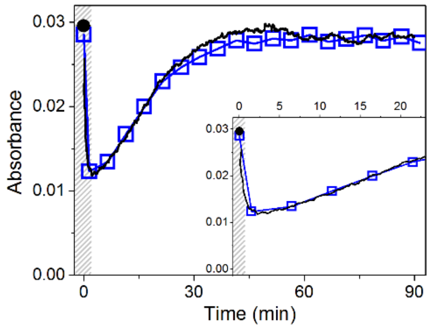

Figure S6. Absorbance changes at 606 nm for 0.50 mM GHK with 0.45 mM Cu(II) in the presence of 1.0 mM GSH measured at a single wavelength with the 5 s intervals (black line, black dot), compared with the readings from spectra collected with 5 minute intervals shifted by 1.5 min (time delay from measurement start to A_{606} recording, blue squares); pH=7.4, T=37 °C; inset depicts changes occurring in the first 20 minutes of the reaction; absorbance values for the binary complex are presented in the shaded field.

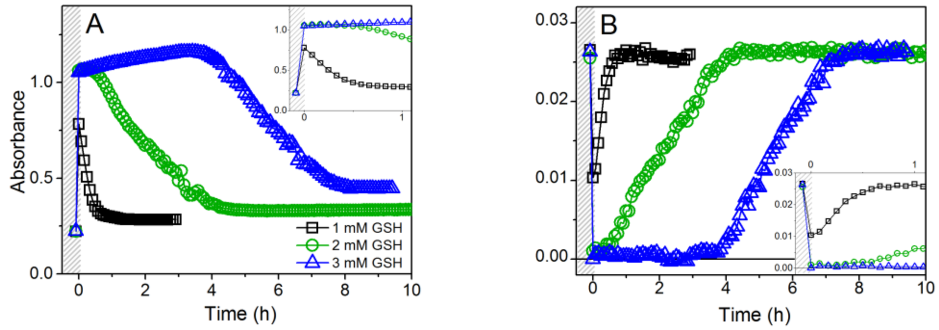

Figure S7. Absorbance changes at (A) 300 nm, (B) 606 nm for 0.50 mM GHK with 0.45 mM Cu(II) in the presence of 1.0 mM (black squares), 2.0 mM (green circles), and 3.0 mM (blue tringles) GSH at pH=7.4, T=37 °C; absorbance changes at 255 nm are not considered as they were out of range for higher GSH concentration; absorbance values for the binary complex are presented in the shaded field; insets depict changes occurring within the first hour of incubation.

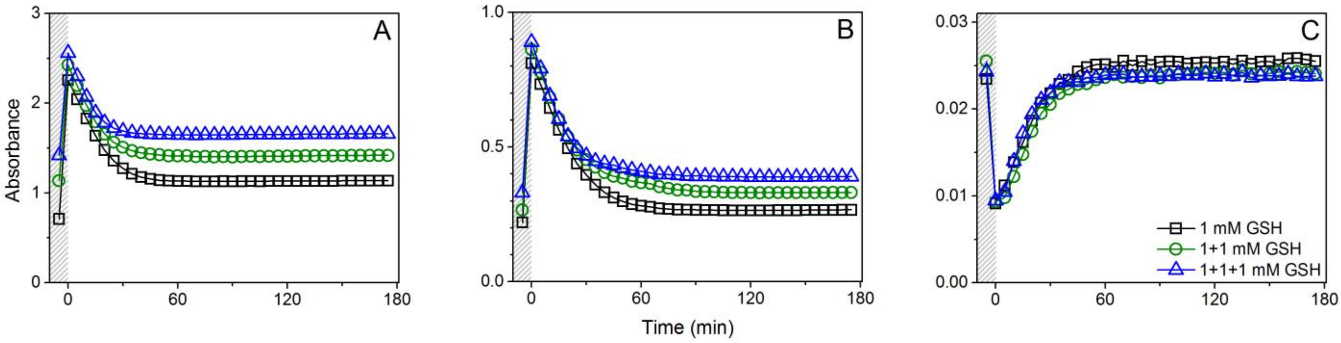

Figure S8 . Absorbance changes at (A) 255 nm, (B) 300 nm, and (C) 606 nm for 0.50 mM GHK and 0.45 mM Cu(II) after recurrent 1 mM GSH additions: the first aliquot (black squares), the second aliquot (green circles), and the third aliquot (blue triangles) of GSH; pH=7.4, T=37°C; absorbance values for the binary complex are presented in the shaded field.

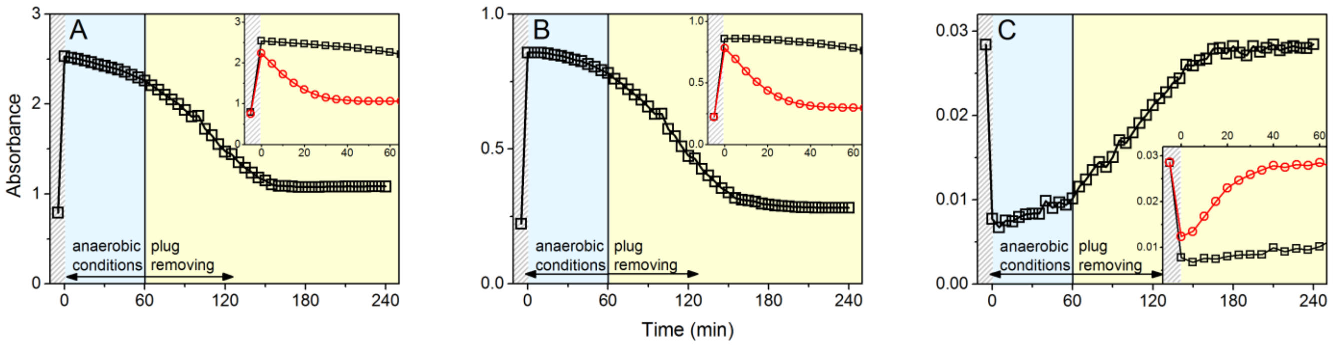

Figure S9. Absorbance changes at (A) 255 nm, (B) 300 nm, and (C) 606 nm for 0.50 mM GHK with 0.45 mM Cu(II) in the presence of 1.0 mM GSH under anaerobic conditions before and after cuvette plug removing; insets show changes occurring within the first hour under anaerobic (black squares) and aerobic (red circles) issue conditions at 37°C, pH=7.4; the oxygen limitation inhibited the recovery of *d-d* band and decline of CT bands (thus confirming the Cu(I) complex stabilization); absorbance value for the binary complex is presented in the shaded field.

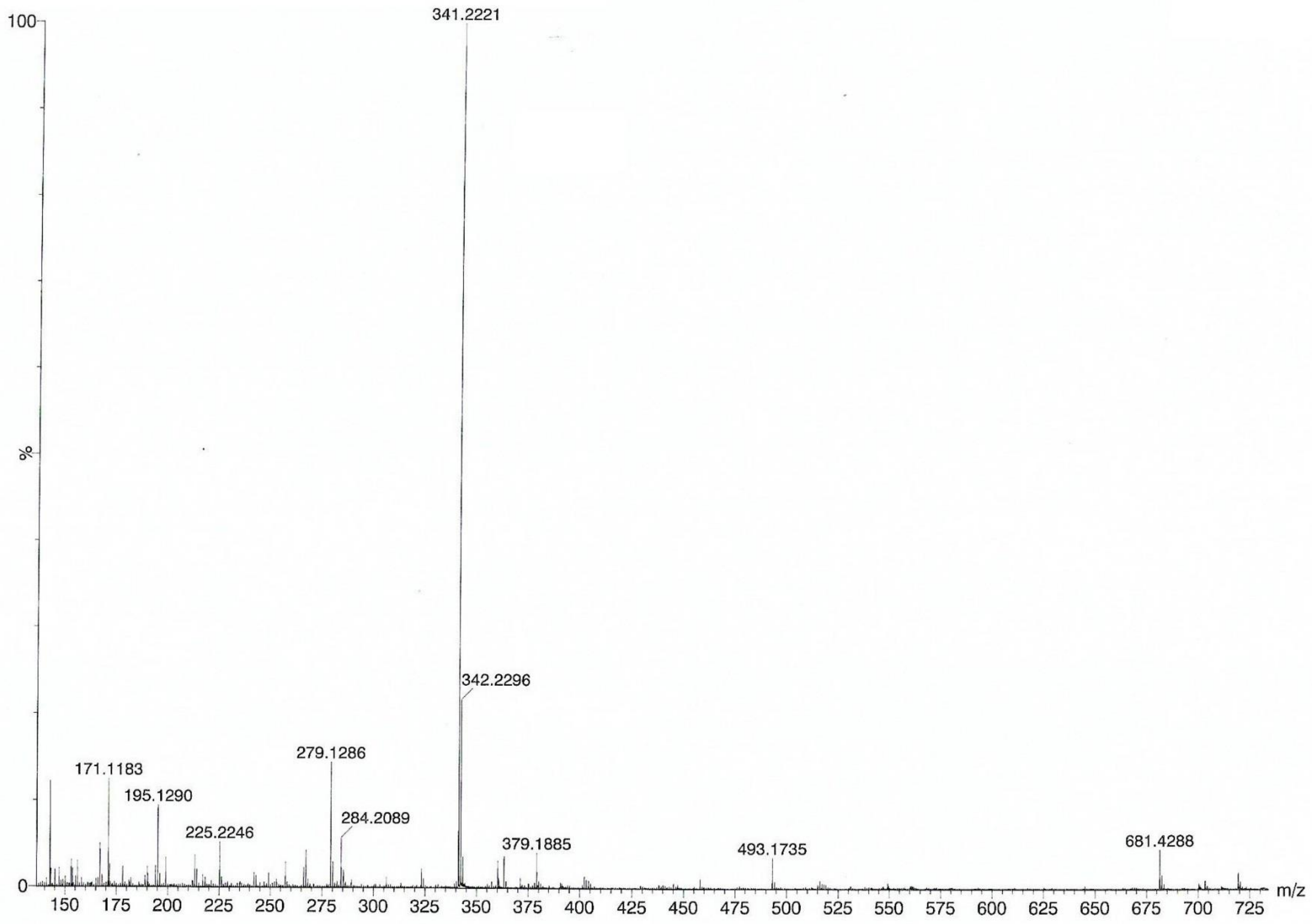

Figure S10. ESI-MS spectra of the fraction containing GHK acquired by HPLC separation of the post-reaction solution.

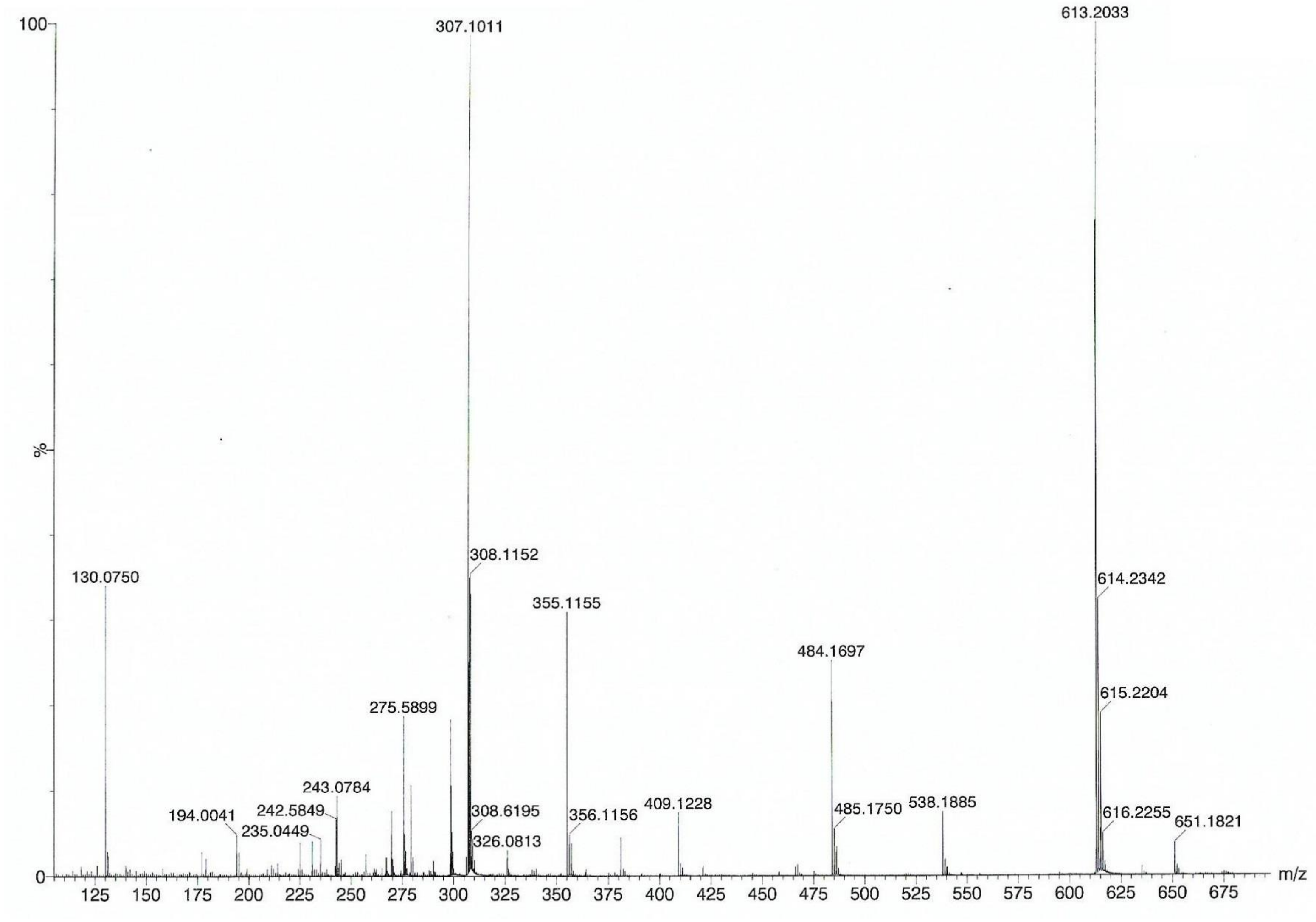

Figure S11. ESI-MS spectra of the fraction containing GSSG acquired by HPLC separation of the post-reaction solution.

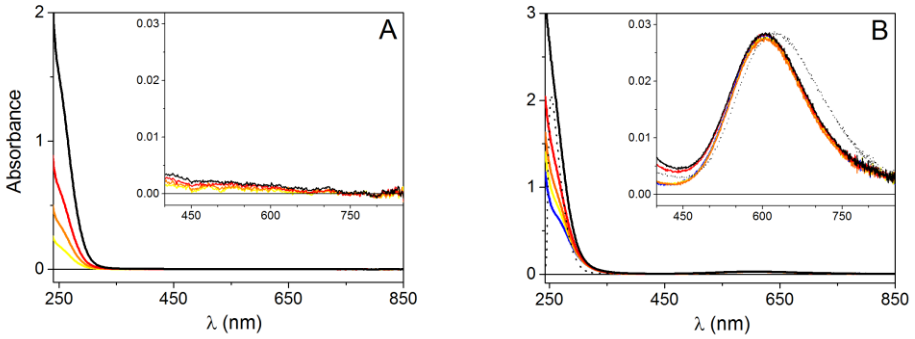

Figure S12. UV-Vis spectra recorded for (A) 0.5 mM (yellow line), 1.0 mM (orange line), 2.0 mM (red line), 5.0 mM (black line) GSSG alone and (B) 0.50 mM GHK with 0.45 mM Cu(II) (blue line) in the presence of corresponding GSSG concentrations (line colours were preserved); the dotted line represents the spectrum of 0.50 mM GSSG with 0.45 mM Cu(II); pH=7.4, room temperature.

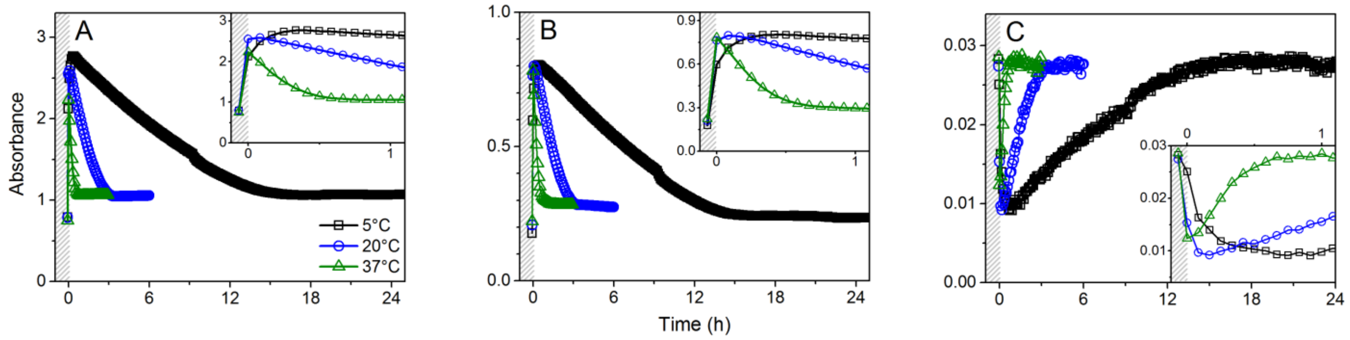

Figure S13. Absorbance changes at (A) 255 nm, (B) 300 nm, and (C) 606 nm for 0.50 mM GHK with 0.45 mM Cu(II) in the presence of 1.0 mM GSH at 5°C (black squares), 20°C (blue circles), and 37°C (green triangles), at pH=7.4; insets depict changes occurring within the first hour; the initial Cu(II)GHK binary complex absorbance is presented in the shaded field.

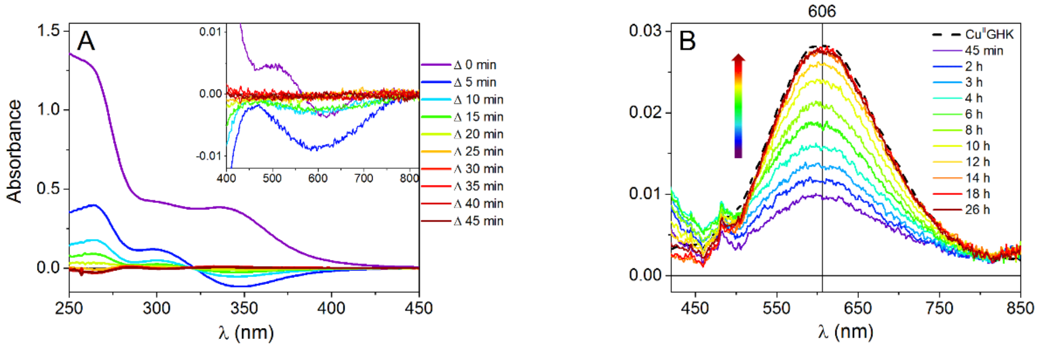

Figure S14. (A) The first 10 differential spectra obtained for 0.50 mM GHK with 0.45 mM Cu(II) in the presence of 1.0 mM GSH at 5°C, pH=7.4; (B) selected UV-Vis spectra from the same experiment (the full set of spectra was collected with 5 min intervals) demonstrating signal restoration; dashed line represents the spectrum of Cu(II)GHK.

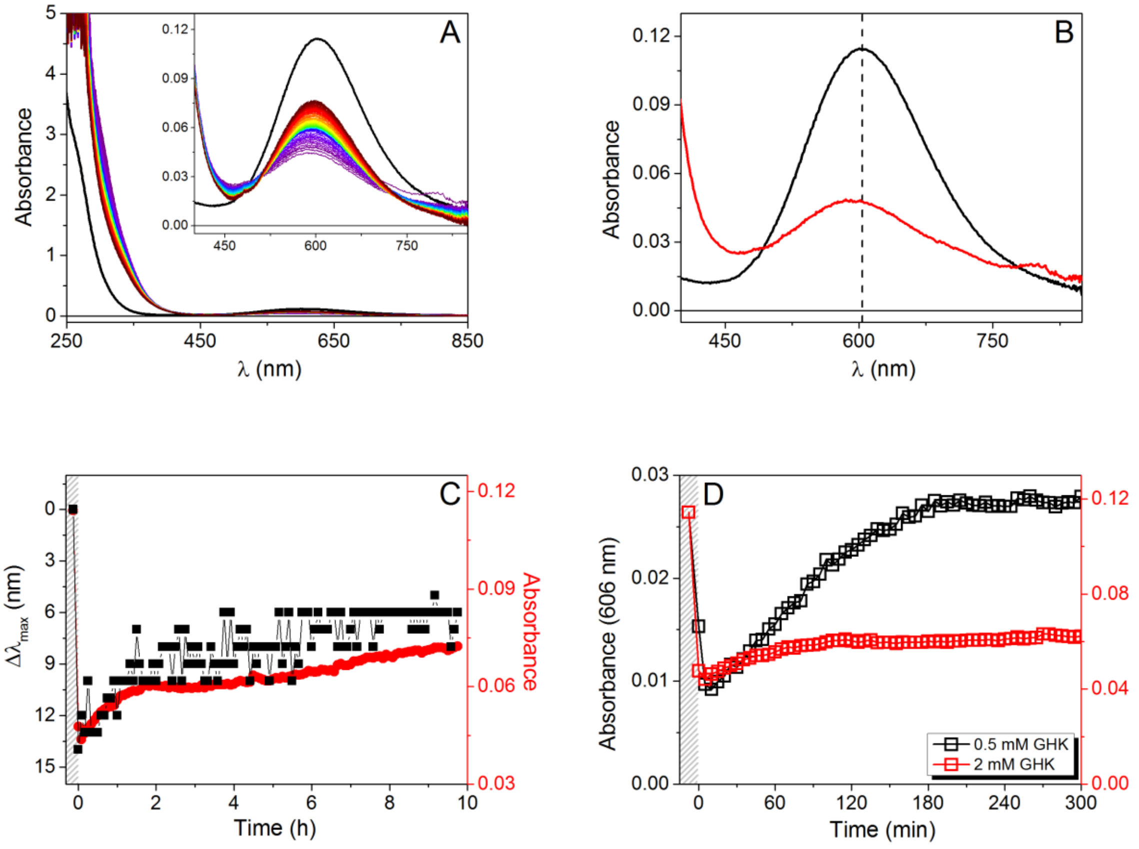

Figure S15. (A) UV-Vis spectra collected with 5 min intervals (for 10 h) for 2.0 mM GHK with 1.80 mM Cu(II) in the presence of 4.0 mM GSH at pH=7.4, T=20 °C, black line represents the initial spectrum of Cu(II)GHK; (B) the initial spectrum of Cu(II)GHK prior to the GSH addition (black line) and the first spectrum after the GSH addition (red line) with the absorbance maximum of Cu(II)GHK marked (dashed line); (C) time-dependent changes in the intensity of the *d-d* band (red circles) in relation to its position (black squares), expressed as a difference between the *d-d* band absorbance maximum read from the spectrum recorded for Cu(II)GHK alone and after the GSH addition; (D) comparison of absorbance changes at 606 nm plotted as a function of time for 2.0 mM GHK with 1.80 mM Cu(II) in the presence of 4.0 mM GSH (red squares) and for 0.5 mM GHK with 0.45 mM Cu(II) in the presence of 1.0 mM GSH (black squares), pH=7.4, T=20°C; absorbance values for the binary complex on panels (C) and (D) are presented in the shaded field.

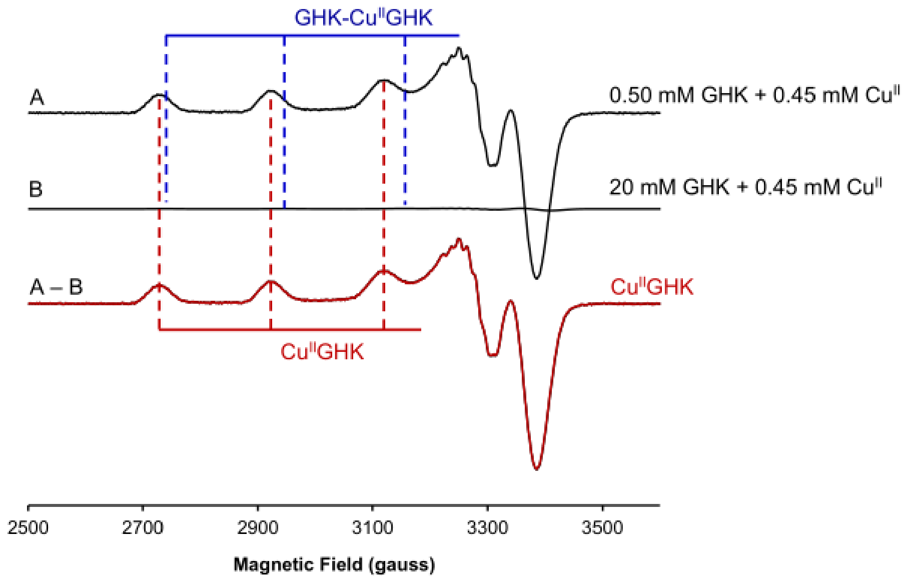

Figure S16. X-band (9.42 GHz) frozen solution (77 K) EPR spectra showing the isolation of the spectrum corresponding to Cu(II)GHK by weighted subtraction of the spectrum of 20 mM GHK with 0.45 mM Cu(II) from 0.50 mM GHK with 0.45 mM Cu(II). Dashed vertical lines indicate the approximate position of the prominent Cu(II) hyperfine features of each coordination mode as a guide to the eye.

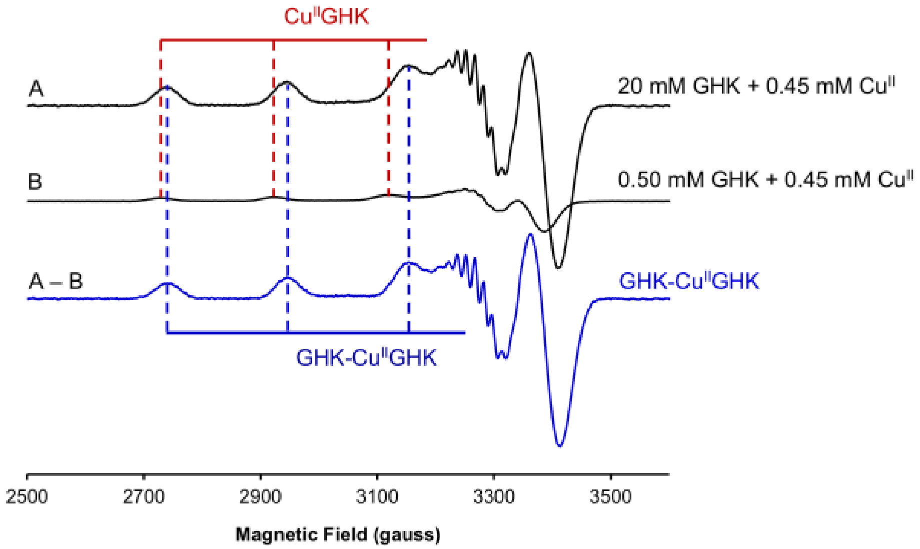

Figure S17. X-band (9.42 GHz) frozen solution (77 K) EPR spectra showing the isolation of the spectrum corresponding to GHK-Cu(II)GHK by weighted subtraction of the spectrum of 0.50 mM GHK with 0.45 mM Cu(II) from 20 mM GHK with 0.45 mM Cu(II). Dashed vertical lines indicate the approximate position of the prominent Cu(II) hyperfine features of each coordination mode as a guide to the eye.

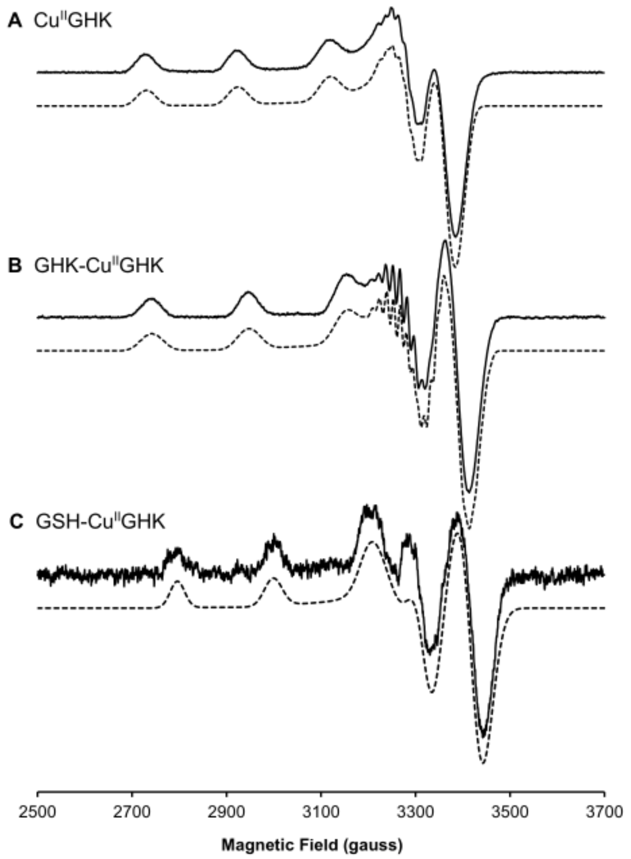

Figure S18. Experimental (———) and simulated (- - - -) frozen-solution EPR spectra of the divalent copper complexes **(A)** Cu(II)GHK, **(B)** GHK-Cu(II)GHK, and **(C)** GSH-Cu(II)GHK using the spin Hamiltonian parameters listed in Table S2.

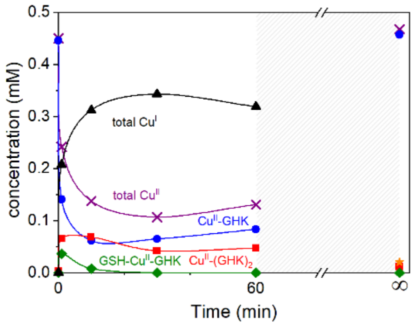

Figure S19. Speciation diagram obtained by decomposition of the time-dependent EPR spectra of 0.50 mM GHK with 0.45 mM Cu(II) in the presence of 1.0 mM GSH (Figure S20); pH=7.4. After 60 minutes of storage on ice, the sample was incubated for yet 2 hours at 37°C (to make sure the reaction is completed), after which the last EPR spectrum was recorded. The total concentration of Cu(II) species was determined by double integration of the first harmonic spectra.

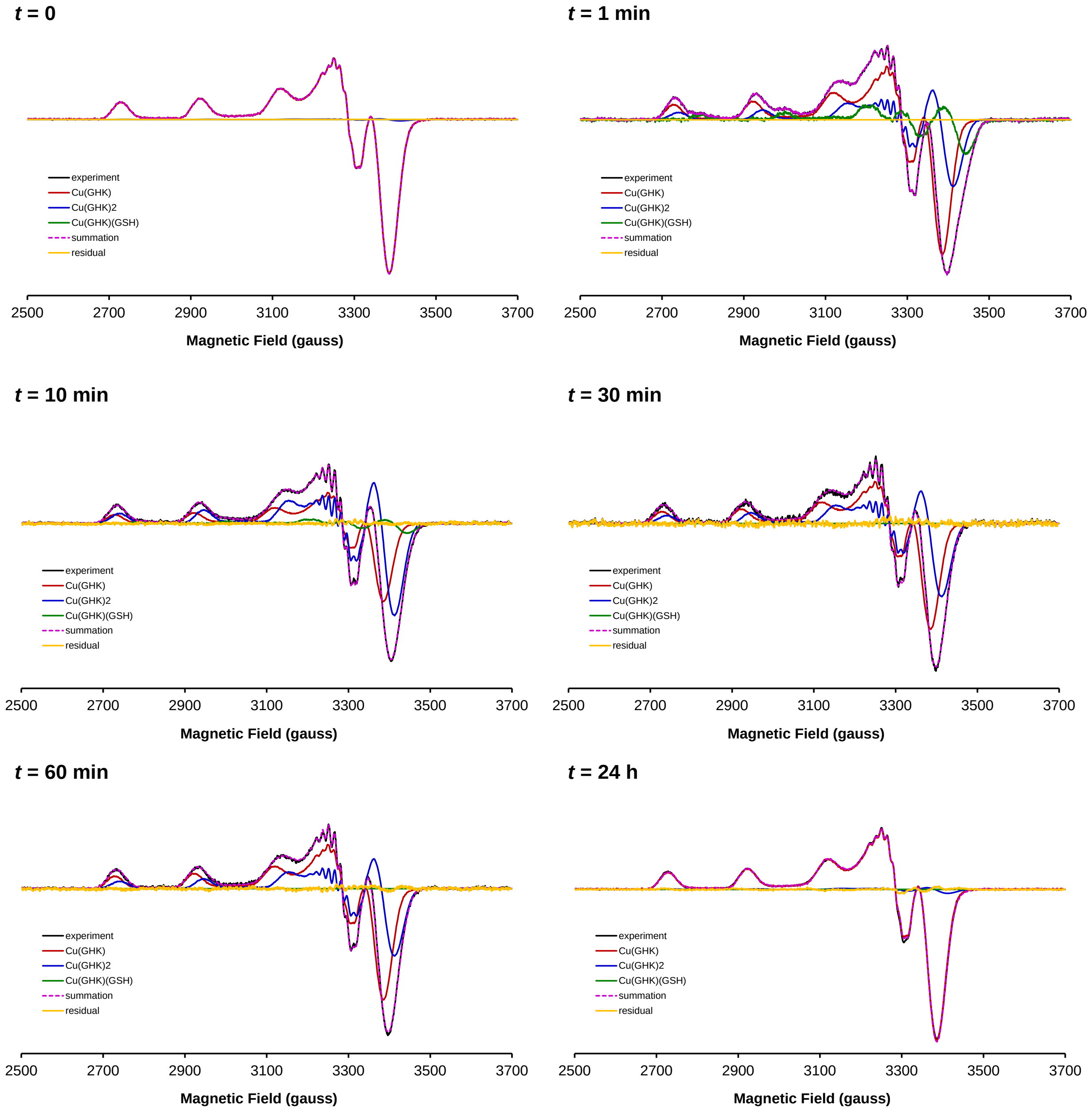

Figure S20. Decomposition of the time-dependent X-band EPR spectra of 0.50 mM GHK with 0.45 mM Cu(II) in the presence of 1.0 mM GSH. The corresponding percentages of each species are shown in Figure S19.

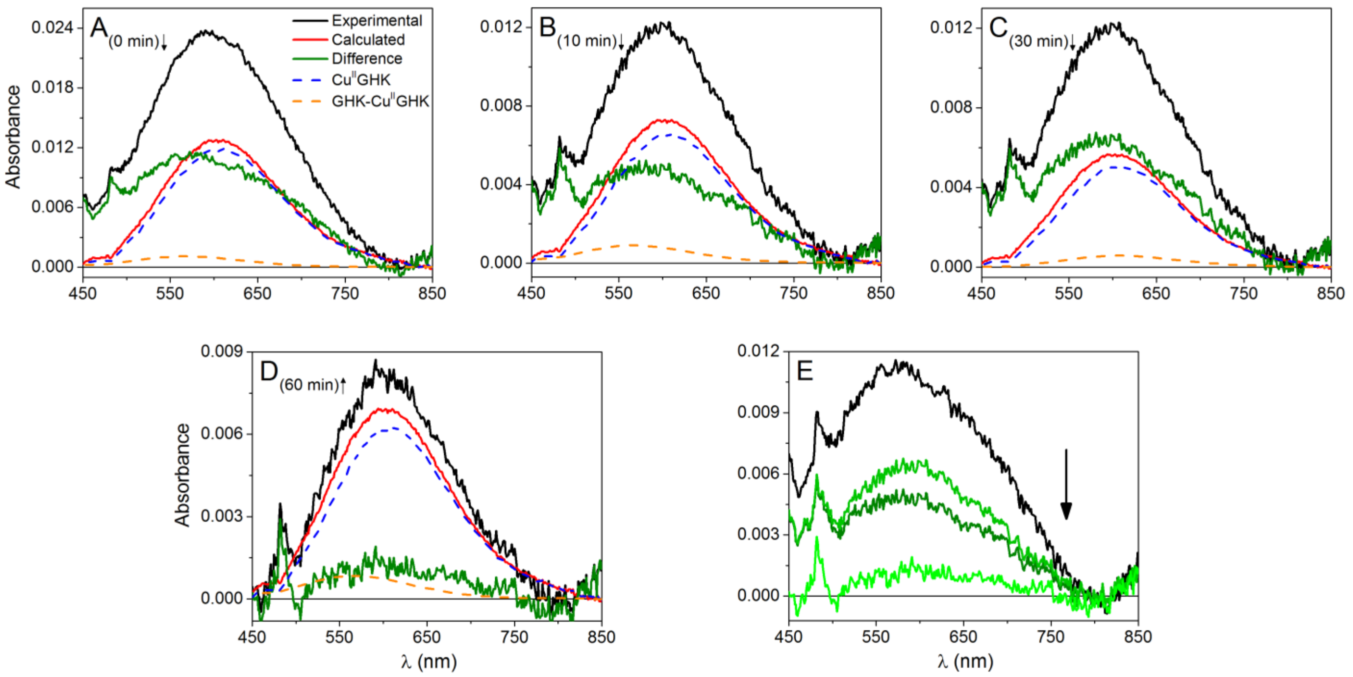

Figure S21 Panels (A-D) depict results of the decomposition of the UV-Vis spectra of 0.50 mM GHK with 0.45 mM Cu(II) at different time points of the reaction with 1.0 mM GSH at 5°C; black line – represents experiment spectrum, red line – calculated one, blue dashed line – Cu(II)GHK binary complex, orange dashed line – Cu(II)-$(GHK)_2$ ternary complex, green line – the difference between experimental and calculated spectrum; panel (E) shows a comparison of the calculated differential spectra; an arrow next to the capital letter indicates on the belongingness of the spectrum under deconvolution to the reduction (↓) or re-oxidation (↑) step; for calculations information about total copper(II) concentration acquired by EPR and literature data on stability constants of binary and auto-ternary GHK complexes[5] were employed.

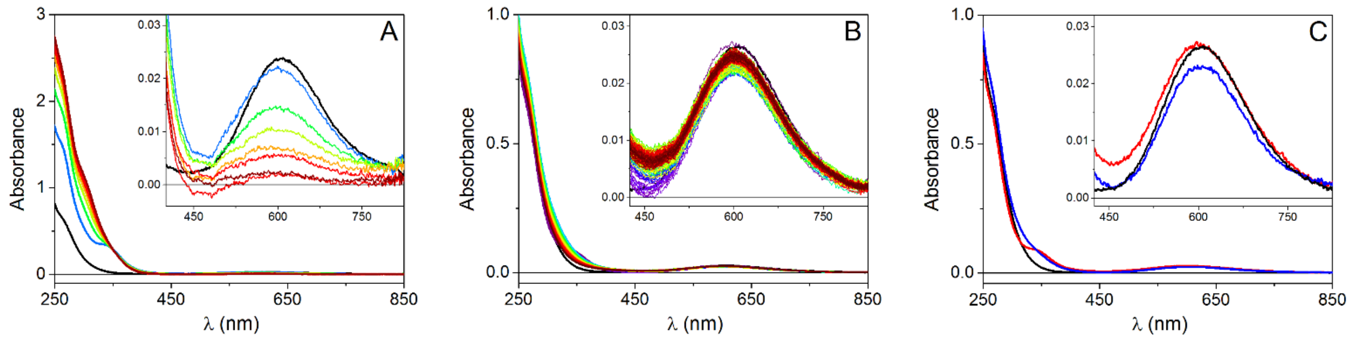

Figure S22. UV-Vis spectra of 0.50 mM GHK with 0.45 mM Cu(II) in the presence of (A) 2.0 mM 2-mercaptoethanol, T=10°C, 5 minutes intervals and (B) 1.0 mM $NaSCH_3$, T=15°C, 10 minute intervals shown for clarity; both at pH=7.4; black line represents the spectrum of the Cu(II)GHK binary complex; panel (C) depicts the first spectrum after $NaSCH_3$ addition (red line) and the one with the lowest absorbance value (blue line).

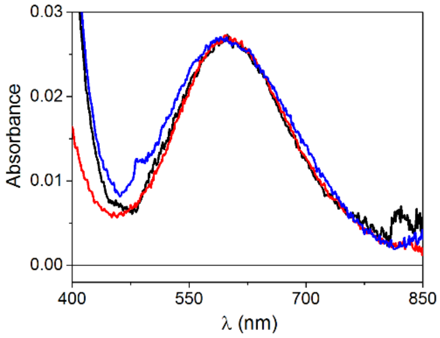

Figure S23. Normalized *d-d* bands spectra of 0.50 mM GHK with 0.45 mM Cu(II) in the presence of 2.0 mM 2-mecaptoethanol, T=10°C (black line), 1.0 mM $NaSCH_3$, T=15°C (red line), 1.0 mM GSH, T=5°C (blue line) (in each case the first recorded spectrum was taken into consideration).

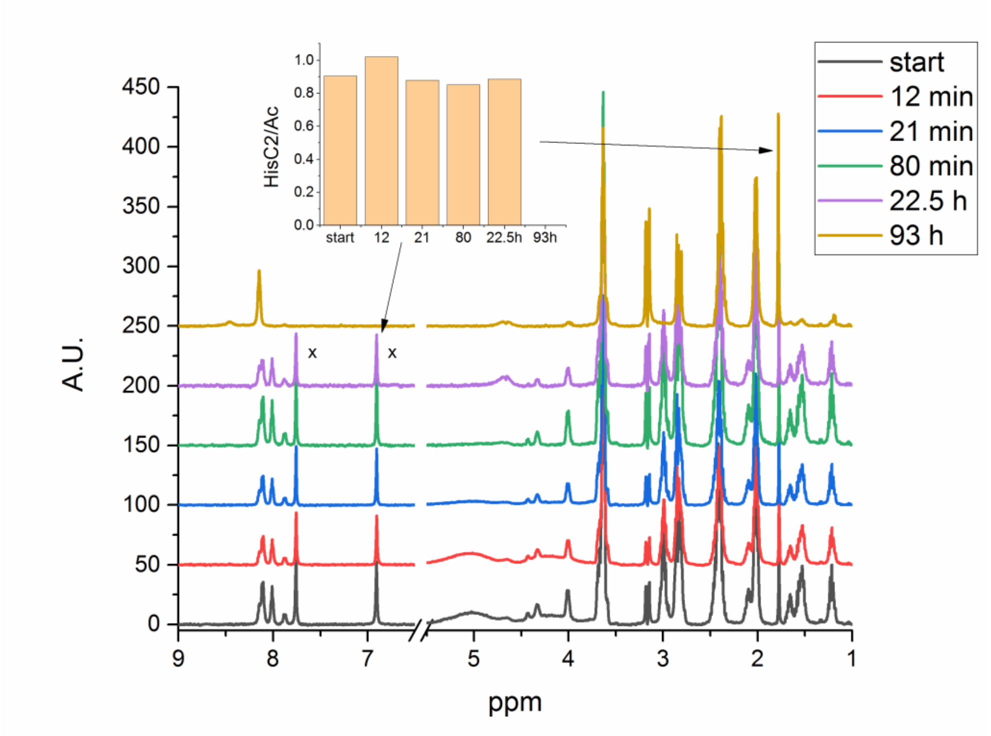

Figure S24. The ^{1}H spectra obtained by adding 2 mM GSH to 0.5 mM GHK with 0.45 mM $Cu(NO_3)_2$ in 50 mM sodium phosphate buffer, at 20°C, pH 7.4,. His imidazole protons of GHK are marked with x. The inset presents the integrals of His C2 proton calibrated against the signal of acetate (the impurity in GHK preparation that does not interact with copper under the applied conditions).

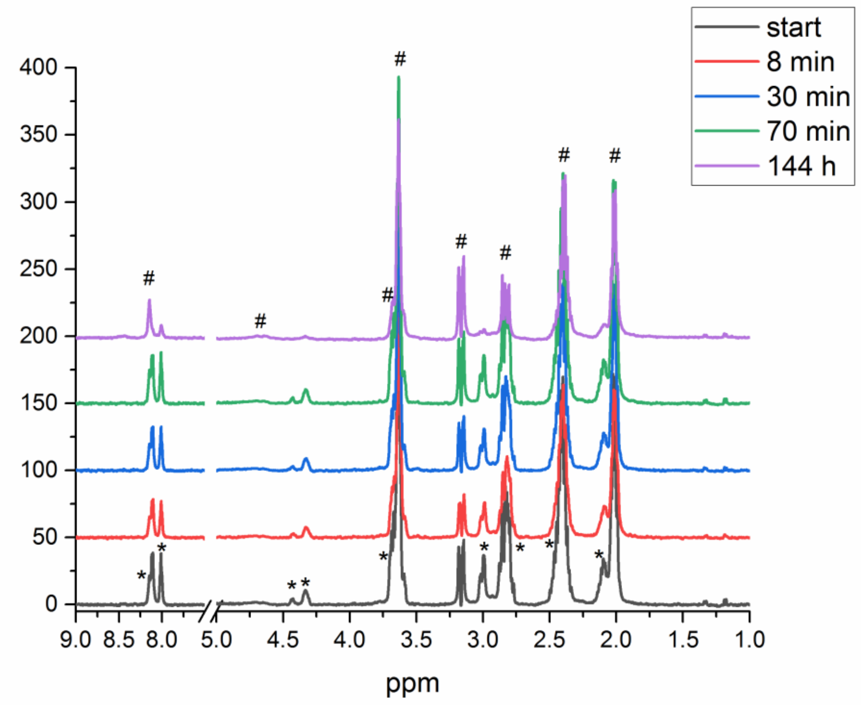

Figure S25. The ^{1}H spectra obtained by adding 0.45 mM $Cu(NO_3)_2$ to 2 mM GSH in 50 mM sodium phosphate buffer, at 20°C, pH 7.4; GSSG peaks are marked with # on the last spectrum; Cu(I)-GSH peaks, which vanish gradually with time, are marked with * on the first spectrum.

Comments: The first four spectra are practically identical and present the mixture of GSSG (#) and Cu(I)-GSH (*), These assignments are supported by the control spectra of 2 mM GSH and 1 mM GSSG apopeptides recorded under the same conditions, which are depicted in Figure S26. The most characteristic signal of Cu(I)-GSH complex is derived from a β proton of Cu(I)-bound Cys, at 3.0 ppm. The last spectrum contains mostly GSSG with only traces of Cu(I)-GSH. All remaining Cu(I) is oxidized back to Cu(II) and complexed with GSSG. The lines of this complex are broadened out by paramagnetic Cu(II), and therefore the spectrum contains the signals from the molar excess of free GSSG.

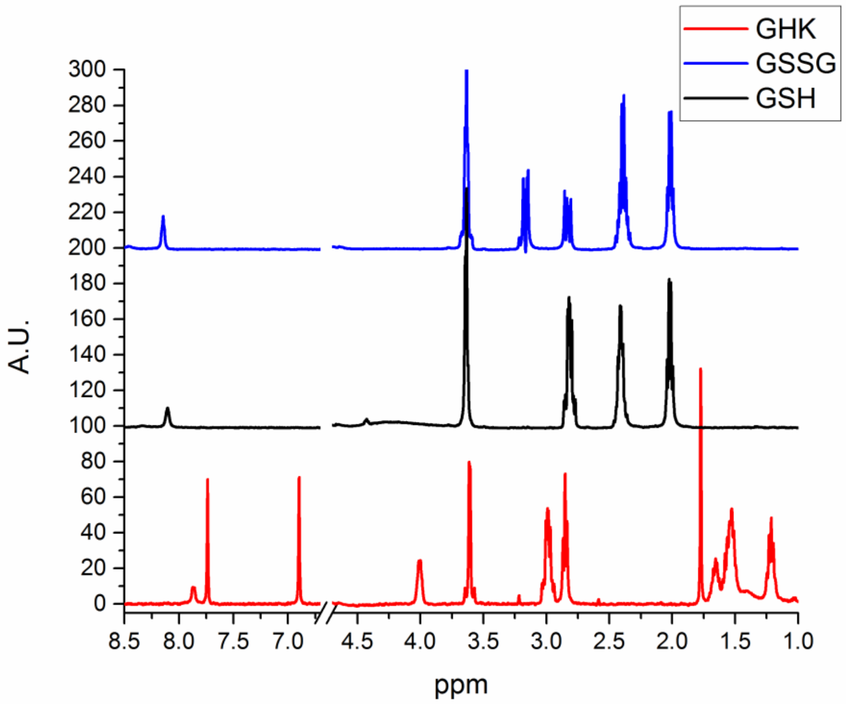

Figure S26. The ^{1}H spectra of 2 mM GSH, 1 mM GSSG, and 0.5 mM GHK, all in 50 mM sodium phosphate buffer, at 20°C, pH 7.4.

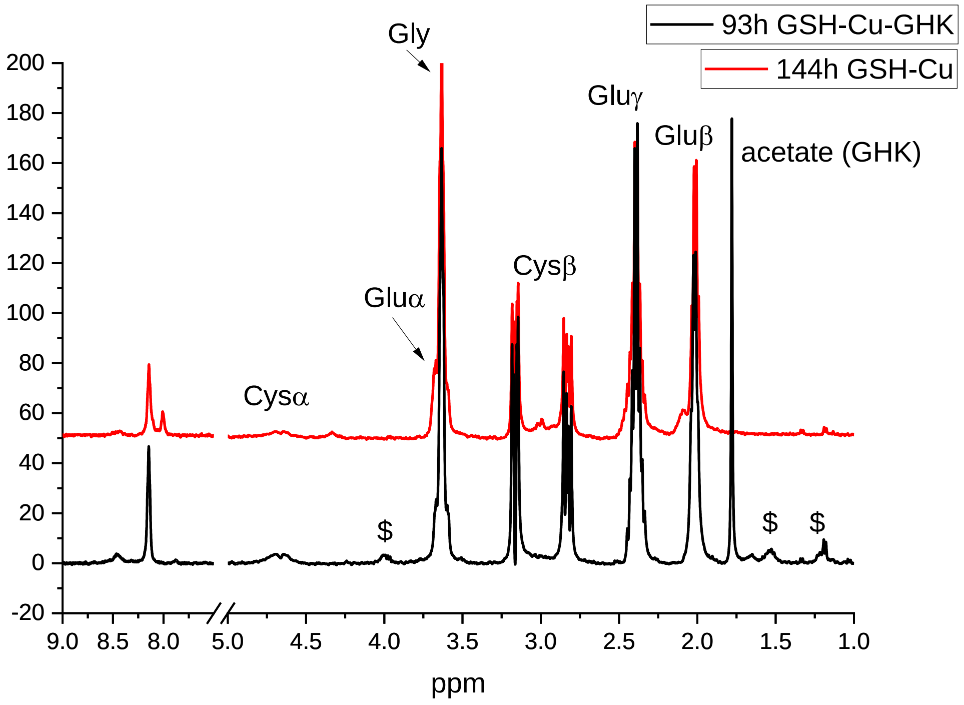

Figure S27. Comparison of final spectra of 2 mM GSH with 0.45 mM Cu(II) recorded at 144 h (red line) and 0.50 mM GHK with 0.45 mM Cu in the presence of 2 mM GSH recorded at 93 h (black line), all in 50 mM sodium phosphate buffer, pH 7.4, 20°C The residual signals of GHK Lys protons are marked with $.

Comments: The final spectra of both samples are identical, except for the acetate signal derived from the GHK formulation, and are dominated by the signals of GSSG. This means that in the presence of GHK, all Cu(II) initially reduced by GSH is reoxidized and bound to GHK, causing broadening out of its signals. Only trace signals of Lys protons of GHK can be noticed at high field.

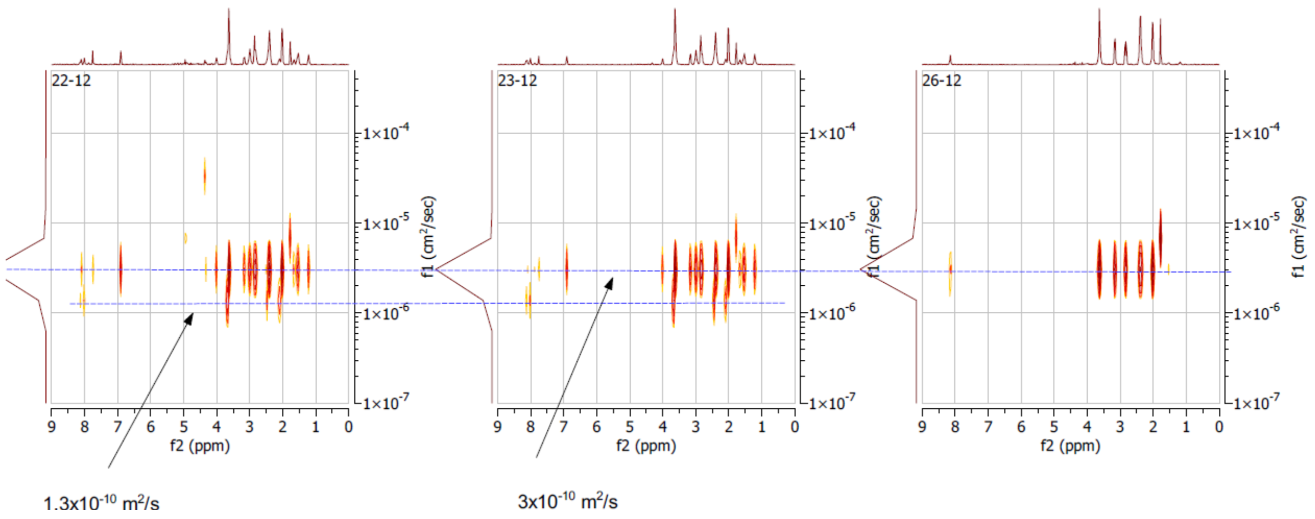

Figure S28. Comparison of DOSY spectra recorded for the selected spectra presented in Figure S24, at the start, 22.5 and 93 h. Lines mark signals exhibiting the same diffusion coefficient.

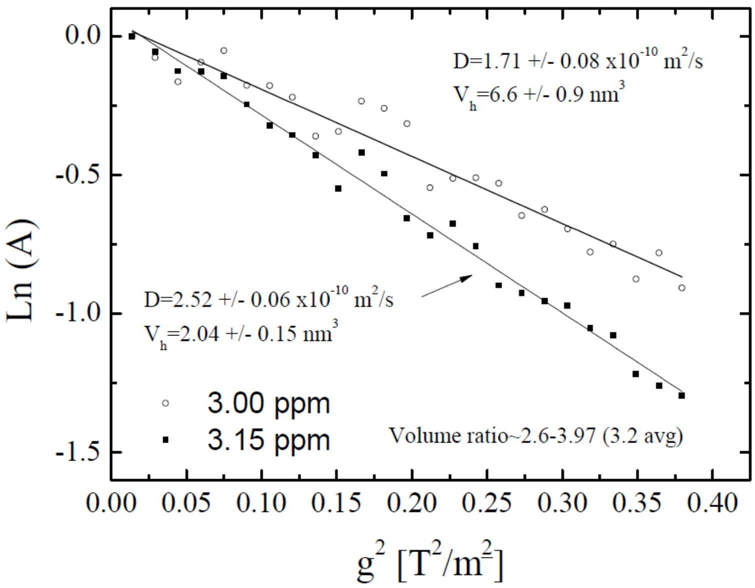

Figure S29. Volume calculation for Cu(I)-GSH and GSSG molecules, based on DOSY signals of their Glu β protons. The specifically obtained D of 2.5 and 1.7 × 10^{-10} $m^2 \cdot s^{-1}$ correspond to the values of 3.0 and 1.3 × 10^{-10} $m^2 \cdot s^{-1}$ from the global analysis presented in Figure S26.

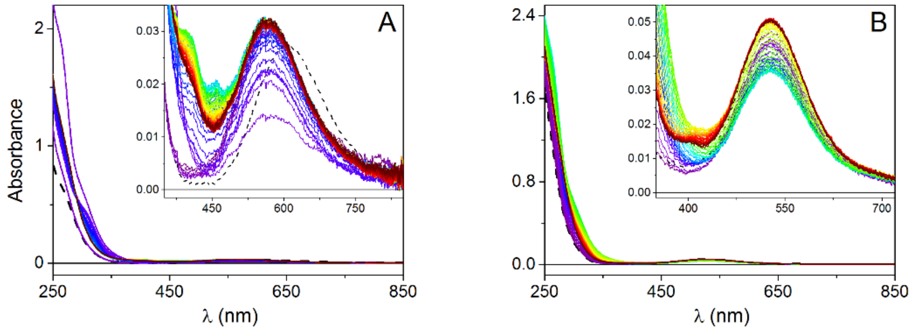

Figure S30. UV-Vis spectra collected with 5 min intervals for (A) 0.50 mM GHK with 0.45 mM Cu(II) and 50 mM imidazole. (B) 0.50 mM GGH with 0.45 mM Cu(II), both in the presence of 1.0 mM GSH. pH=7.4. T=37°C; dashed line represents the spectrum of the corresponding binary complex.

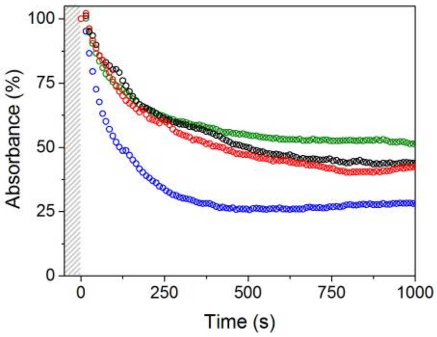

Figure S31. Time-wise absorbance changes at a single wavelength presented as a percentage of the initial absorbance value (obtained as a result of extrapolation to zero time point) for 0.50 mM GHK with 0.45 mM Cu(II) in the presence of 1.0 mM GSH: without imidazole (blue circles) and with 10 mM (green circles), 30 mM (black circles) and 50 mM (red circles) imidazole; all points were collected with 10 s intervals at λ_{max} (565 nm and 606 nm for experiments with and without imidazole, respectively), pH=7.4, T=20°C.

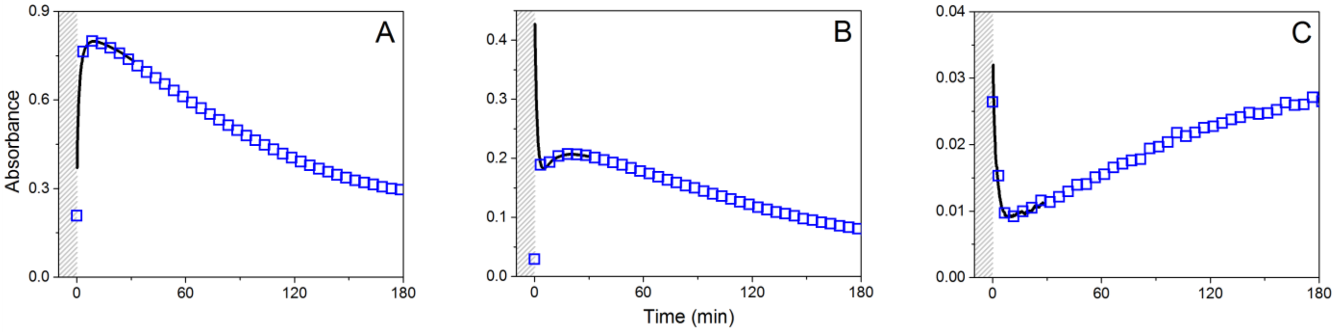

Figure S32 Absorbance changes at (A) 300 nm (B) 345 nm and (C) 606 nm for 0.50 mM GHK with 0.45 mM Cu(II) in the presence of 1.0 mM GSH at 20°C, pH=7.4; solid line represents measurement at one wavelength (5 s intervals), blue squares are the readings from spectra collected with 5 minute intervals (shifted by the real time of particular wavelength measurement); absorbance values for the binary complex are marked as 0 time points.

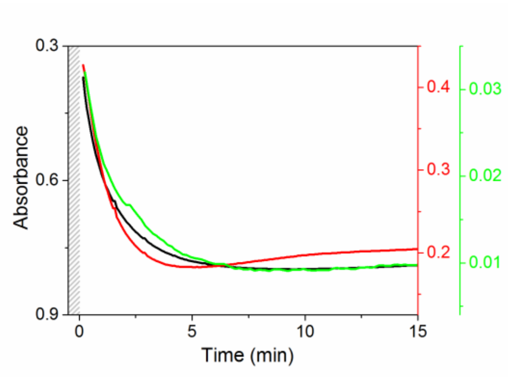

Figure S33 Absorbance changes at 300 nm are shown in inverted scale for the purpose of comparison (black line)with 345 nm (red line) and 606 nm (light green line) for 0.50 mM GHK with 0.45 mM Cu(II) in the presence of 1.0 mM GSH at 20°C, pH=7.4.

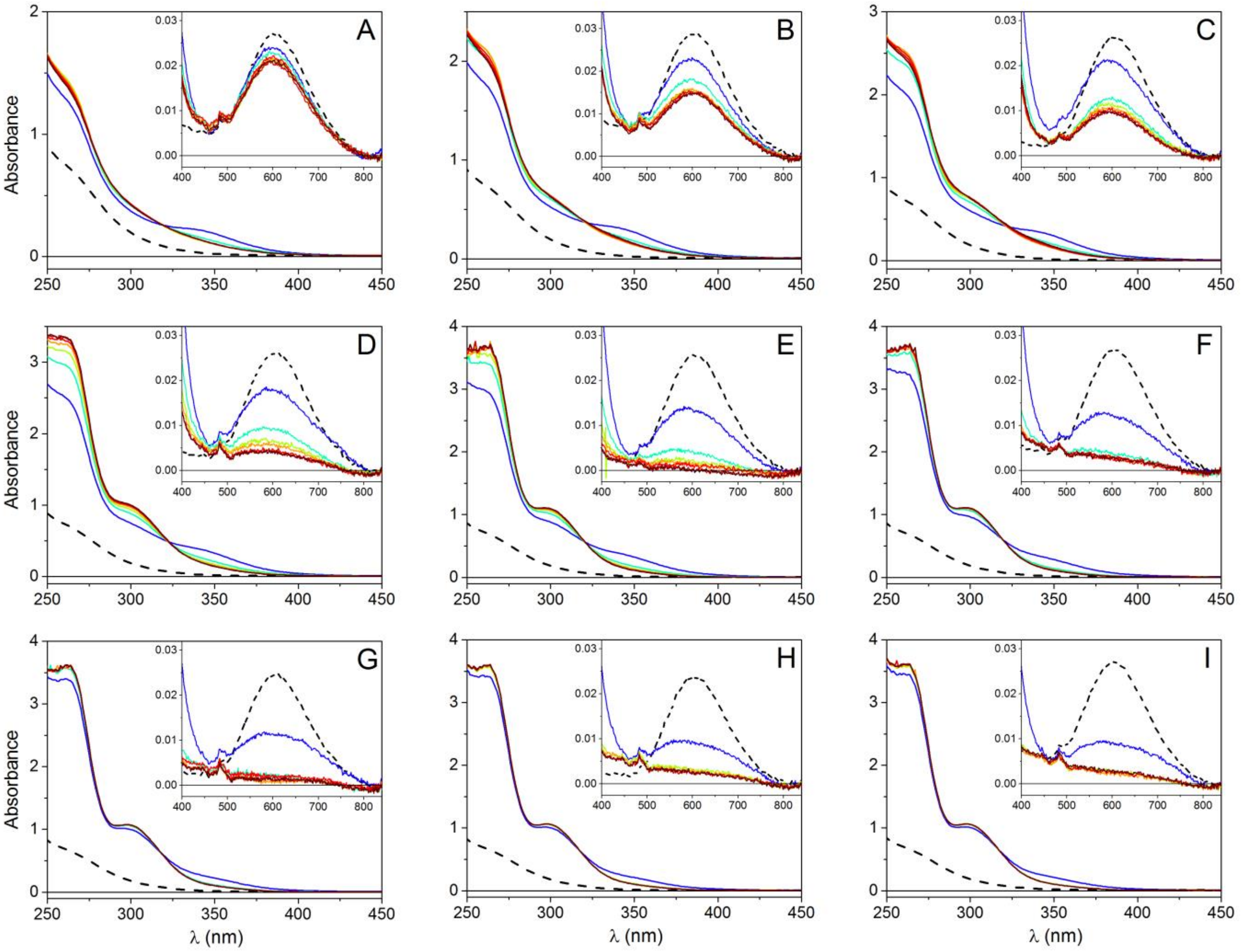

Figure S34. UV-Vis spectra collected with 5 min intervals for 0.50 mM GHK with 0.45 mM Cu(II) in the presence of GSH with a concentration of (A) 0.4 mM. (B) 0.8 mM. (C) 1.0 mM. (D) 1.5 mM. (E) 2.0 mM. (F) 3.0 mM. (G) 5.0 mM. (H) 7.5 mM. (I) 10.0 mM; pH=7.4. T=5°C; dashed line represents the spectrum of the Cu(II)GHK binary complex; due to the distinct contribution of Cu(II)GHK absorption in the UV range. The binary complex signal was subtracted from all spectra representing the progress of the reaction with GSH.

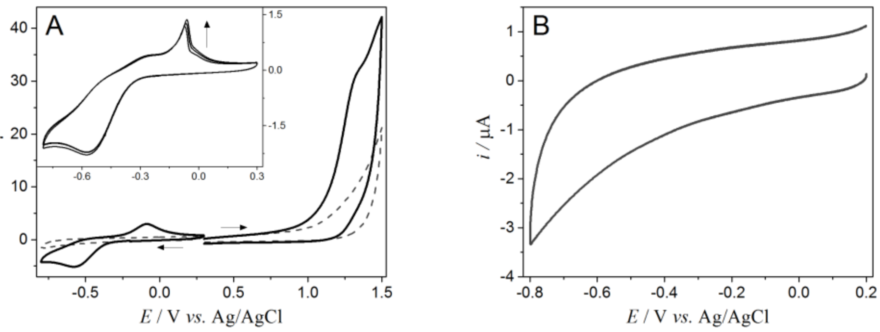

Figure S35. CV curves recorded for (A) 0.50 mM GHK in the absence (dashed line) and presence of 0.45 mM Cu(II) (solid line), ʋ = 100 mV/s; inset represents the current response for Cu(II)GHK complex obtained in the given range of potentials at the 20 mV/s scan rate; (B) CV curve of 1.0 mM GSH with 0.45 mM Cu(II), ʋ = 100 mV/s; electrochemical experiments were conducted in 100 mM KNO_3 at room temperature, pH 7.4.

References

(1) Postal, W. S.; Vogel, E. J.; Young, C. M.; Greenaway, F. T. The Binding of Copper(II) and Zinc(II) to Oxidized Glutathione. *J. Inorg. Biochem.* **1985**, *25* (1), 25–33. https://doi.org/10.1016/0162-0134(85)83004-X.

(2) Wishart, D. S.; Bigam, C. G.; Yao, J.; Abildgaard, F.; Dyson, H. J.; Oldfield, E.; Markley, J. L.; Sykes, B. D. 1H, 13C and 15N Chemical Shift Referencing in Biomolecular NMR. *J. Biomol. NMR* **1995**, *6* (2), 135–140. https://doi.org/10.1007/BF00211777.

(3) Stejskal, E. O.; Tanner, J. E. Spin Diffusion Measurements: Spin Echoes in the Presence of a Time-Dependent Field Gradient. *J. Chem. Phys.* **1965**, *42* (1), 288–292. https://doi.org/10.1063/1.1695690.

(4) Sugiura, Y. Proton-Magnetic-Resonance Study of Copper(I) Complexes with Peptides Containing Sulfhydryl and Imidazole Groups as Possible Model Ligands for Copper Proteins. *Eur. J. Biochem.* **1977**, *78* (2), 431–435. https://doi.org/10.1111/j.1432-1033.1977.tb11755.x.

(5) Bossak-Ahmad, K.; Wiśniewska, M. D.; Bal, W.; Drew, S. C.; Frączyk, T. Ternary Cu(II) Complex with GHK Peptide and Cis-Urocanic Acid as a Potential Physiologically Functional Copper Chelate. *Int. J. Mol. Sci.* **2020**, *21* (17), 6190. https://doi.org/10.3390/ijms21176190.
